# TIM-3 Expression Is Downregulated on Human NK Cells in Response to Cancer Targets in Synergy with Activation

**DOI:** 10.3390/cancers12092417

**Published:** 2020-08-26

**Authors:** Tram N. Dao, Sagar Utturkar, Nadia Atallah Lanman, Sandro Matosevic

**Affiliations:** 1 Department of Industrial and Physical Pharmacy, Purdue University, West Lafayette, IN 47907, USA; dao2@purdue.edu; 2 Center for Cancer Research, Purdue University, West Lafayette, IN 47907, USA; sutturka@purdue.edu (S.U.); natallah@purdue.edu (N.A.L.); 3 Department of Comparative Pathobiology, Purdue University, West Lafayette, IN 47907, USA

**Keywords:** TIM-3, NK cell receptor, immunotherapy, solid tumor, NK cell activation

## Abstract

Among natural killer (NK) cell receptors, the T-cell immunoglobulin and mucin-containing domain (TIM-3) has been associated with both inhibitory and activating functions, depending on context and activation pathway. Ex vivo and in vitro, expression of TIM-3 is inducible and depends on activation stimulus. Here, we report that TIM-3 expression can be downregulated on NK cells under specific conditions. When NK cells are exposed to cancer targets, they synergize with stimulation conditions to induce a substantial decrease in TIM-3 expression on their surface. We found that such downregulation occurs following prior NK activation. Downregulated TIM-3 expression correlated to lower cytotoxicity and lower interferon gamma (IFN-γ) expression, fueling the notion that TIM-3 might function as a benchmark for human NK cell dysfunction.

## 1. Introduction

It is increasingly evident that modulation of immune-regulatory receptors in the tumor microenvironment in favor of restored anti-tumor immunity is becoming one of the pivotal strategies in the adaptation of cell-based immunotherapies to target various cancers [1].

Natural killer (NK) cell effector responses are guided by a controlled balance of inhibitory and activating receptors expressed on the NK cell surface, and their interactions with cognate ligands on target cells [2]. Among NK cell receptors, T-cell immunoglobulin and mucin-containing domain (TIM-3), also known as hepatitis A virus cellular receptor 2, has been characterized as both: its role has been linked to promoting both inhibitory and activating functions [3,4], in addition to characterizing either a mature or exhausted NK phenotype [5]. Notably, studies have shown that TIM-3 can impair NK cell cytotoxicity [3], or enhance interferon gamma (IFN-γ) production [4]. Pires da Silva and colleagues [6] described TIM-3 as an inhibitory receptor, and reported its upregulation on NK cells from the blood of melanoma patients to correlate to these cells’ functional exhaustion. Blocking TIM-3 partially rescued these patient-derived NK cells’ anti-tumor cytotoxicity [6]. As a marker of maturation, expression of TIM-3 has been shown to correlate with the terminally mature CD56^dim^CD16^+^ NK phenotype [3]. Clinically, TIM-3 expression on NK cells from patients with various cancers has been associated with disease progression and poor prognosis. Data have evidenced such TIM-3^+^ NK cells in lung cancer [7], bladder cancer [8,9], gastric cancer [10], esophageal cancer [11], endometrial cancer [12], melanoma [13] and, to an extent, glioma [14]. Findings in cancer do not necessarily correlate to other diseases, for instance autoimmune disorders, where decreased expression of TIM-3 on NK cells was shown to correlate to better prognosis [15].

TIM-3 expression is not unique to NK cells, as this receptor is also expressed on cytotoxic T lymphocytes, macrophages, dendritic cells and mast cells, among others [16,17]. On T cells in tumors, TIM-3 expression was correlated to dysfunctional anti-tumor immunity [18]. Though TIM-3 blockade was shown to result in enhanced T cell-induced IFN-γ production [19], studies have suggested a superior therapeutic effect when TIM-3 blockade was combined with programmed cell death protein 1 (PD-1) inhibition [20]. This was buoyed by discoveries indicating that expression of TIM-3 and PD-1 was positively correlated on tumor-infiltrating lymphocytes in a variety of solid tumors [21]. Additionally, resistance to anti-PD-1 monotherapy was correlated with the upregulation of TIM-3 in mouse models of lung adenocarcinoma [22]. Similar findings have been mirrored on NK cells, with studies having demonstrated co-expression of PD-1 and TIM-3 on NK cells from patients with various cancers [23,24].

The role of TIM-3 on NK cells has been defined by its ability to be induced by various stimuli, including cytokines and culture conditions. Notably, TIM-3 could be induced in vitro by the cytokines IL-2, IL-12, IL-15, IL-18 and IFNα as well as Fc receptor (FcR) interactions [3,13,25]. Despite the inducible upregulation of TIM-3 expression, TIM-3 is constitutively expressed on NK cells in the native state [3]. In vitro, overexpression of TIM-3 on NK cells from healthy donors, stimulated with IL-2, was shown to give rise to highly cytotoxic cells with the highest IFN-γ production [6]. That the activation stimulus drives the extent of the effect of TIM-3 expression on NK cell function was evidenced further by So and colleagues [25], who showed that TIM-3 overexpression resulted in enhanced CD107a production by NK cells when exposed to K562 targets, while exposure to immunoglobulin G (IgG) Fc multimer GL-2045 alone yielded no IFN-γ and low CD107a.

Here, we present a previously unreported finding: expression of TIM-3 can be downregulated on NK cells in response to cancer targets (glioblastoma and prostate cancer) under certain stimulation conditions. Such downregulation occurs under specific stimulation parameters and is unique to TIM-3 rather than other NK cell receptors. We find that NK cells with downregulated TIM-3 are less cytotoxic, and TIM-3 blockade is unable to restore impaired function. Bioinformatics analysis of patient data further revealed correlation between TIM-3 expression in cancer and NK function. These results point to TIM-3 being an inducible marker of functional maturity or exhaustion on NK cells in vitro, urge caution when using TIM-3 as a predictive marker of NK cell status, and highlight the importance of stimulation conditions in the preparation of these cells for adoptive transfer immunotherapy.

## 2. Materials and Methods

### 2.1. Isolation and Purification of Human NK Cells from Peripheral Blood

Fresh whole blood (approximately 100 mL per donor) was obtained from healthy adult volunteer donors under approval by Purdue University’s Institutional Review Board (IRB). NK cells were isolated by immunomagnetic negative selection using the EasySep Direct Human NK Cell Isolation Kit (StemCell Technologies). The cells were expanded at 37 °C and 5% CO_2_ in either CTS OpTmizer™ T Cell Expansion medium (ThermoFisher, A1048501), henceforth referred to as OpTmizer™, or the in-house RPMIf medium. The complete OpTmizer™ medium was supplemented with 2.6% OpTmizer™ supplement, 5% human AB (hAB) serum (Akron Biotech, AK9905), 1% penicillin/streptomycin (Pen/strep) (Gibco, 15140122), 0.2 mM l-glutamine (Gibco, 25030081), 10 ng/mL rhIL-15 (Shenandoah, 100-86), 500 IU/mL rhIL-2 (Akron Biotech, AK8227), and 25 ng/mL rhIL-21 (Gold Biotechnology, 1110-21-2). The RPMIf medium consisted of RPMI 1640 (Gibco, 11875085), 10% fetal bovine serum (FBS) (Corning, 35-016-CV), 1% Pen/strep, 50 ng/mL 4-1BBL (Peprotech, 310-11), 500 IU/mL rhIL-2, 50 ng/mL rhIL-21. The RPMIf expansion medium is commonly used for the expansion of NK cells ex vivo, largely due to the high rates of expansion that such a medium is able to achieve when feeder cells are present. Feeder cells were K562, chemically treated with 50 µg/mL mitomycin C (Cayman Chemical, 11435) for three hours.

### 2.2. Cell Culture

NK cells were first expanded in RPMIf medium and transferred to new medium as specified, culturing in the new medium for at least 48 h before assays. Uppsala 87 Malignant Glioma (U87MG) cells (obtained from Dr. Karen Pollok, Indiana University School of Medicine) were cultured in IMDM 1× (Gibco, 12440046) + 10% FBS + 1% Pen/strep. Prostate cancer (PC)3 cells (Obtained from Dr. Marxa Figueiredo, Purdue University) were cultured in DMEM 1× with Sodium Pyruvate (Gibco, 11995065) + 10% FBS + 1% Pen/strep. Glioblastoma (GBM)43 cells (obtained from Dr. Karen Pollok, Indiana University School of Medicine) were cultured in DMEM 1× without Sodium Pyruvate (Gibco, 11965092) + 10% FBS + 1% HEPES (Gibco, 15630080). All cell cultures were kept at 37 °C and 5% CO_2._

### 2.3. Evaluation of NK Cell Receptor Expression

Target cells (U87MG, PC3 or GBM43) were allowed to attach in wells for at least 6 h. Then, wells were washed once with 1× PBS, and effector NK cells cultured in OpTmizer™ were added at effector:target (E:T) ratios of 10:1 and 2.5:1. After co-incubation for 4 h, cells were collected and stained for flow cytometry. For U87-MG, the entire receptor repertoire was evaluated [PD1, natural killer group 1 a (NKG2A), TIM-3, lymphocyte-activation gene 3 (LAG-3), CD158b, carcinoembryonic antigen-related cell adhesion molecule 1 (CEACAM-1)]. For PC3 and GBM43, TIM-3 expression was evaluated.

### 2.4. Evaluation of Cytokine, Media and Supplement Stimulation of NK Cells

GBM43 cells were allowed to attach in wells for at least 6 h. Then, wells were washed once with 1× PBS, and effector NK cells cultured in either RPMIf, OpTmizer™ or one of the media recipes as listed in Table 1 were added at E:T ratio of 2.5:1. Resting NK cells were cultured in RPMIf or OpTmizer™ without cytokines and OpTmizer™ supplement overnight. After co-incubation of 4 h, cells were collected and stained for flow cytometric evaluation of TIM-3 expression.

### 2.5. Cytotoxicity Assay

GBM43 target cells were labeled with carboxyfluorescein succinimidyl ester (CFSE) for 20 min at 37 °C using the 7-AAD/CFSE Cell-Mediated Cytotoxicity Assay Kit (Cayman Chemical, Ann Arbor, MI, USA), and were allowed to attach in wells for at least 6 h. Then, wells were washed with 1× PBS, and effector NK cells cultured in either RPMIf or OpTmizer™ were added at E:T ratio of 2.5:1. For blockade assays, NK cells were treated with 10 µg/mL anti-TIM-3 blocking antibody (anti-TIM-3 Ab) (Biolegend, LEAF^TM^ clone F38 2E2, San Diego, CA, USA) overnight before addition to target cells. After co-incubation of 4 h, cells were collected, stained with 7-AAD for 15 min at 4 °C, and resuspended in FACS buffer for flow cytometry. Dead target cells are 7-AAD^+^/CFSE^+^.

### 2.6. Flow Cytometry

For extracellular surface staining, samples were collected with 0.25% Trypsin/EDTA (Gibco, 25200056), washed twice with FACS buffer (1× PBS, 5% FBS) and stained with antibodies against CD56-PECy5.5 (Invitrogen, 35-0567-42, Carlsbad, CA, USA) and CD3-PECy7 (BD Biosciences, 560910, San Jose, CA, USA) for identification of NK cells. The following antibodies were used for receptor evaluation: PD-1-BV421 (BD Biosciences, 564323), NKG2A-PE (R&D Systems, FAB1059P025, Minneapolis, MN, USA), TIM-3-APC (Invitrogen, LS17310942), LAG-3-BV421 (BD Biosciences, 565720), CD158b-PE (Biolegend, 312606), CEACAM-1-APC (Invitrogen, 17066180). Samples were stained for 30 min at 4 °C in the dark, washed once with FACS Buffer, and stained with Sytox Green Dead Cell Stain (ThermoFisher, S34860, Waltham, MA, USA) before data acquisition. For intracellular staining, samples were first stained with Live/Dead Fixable Green Dead Cell Stain (ThermoFisher, L23101), then surface stained with CD56-PECy5.5 and CD3-PECy7. Fixation and permeabilization were performed with Cytofix-Cytoperm kit (BD Biosciences, 554714) according to the manufacturer’s instructions. After permeabilization, cells were stained with IFNγ-APC (Invitrogen, 17731982), and resuspended in FACS Buffer for data acquisition. All flow cytometry data were collected on a BD Fortessa and analyzed via FlowJo V10 software (BD, Ashland, OR, USA). Gating strategy for TIM-3 expression on CD56^+^CD3^−^ NK cells is shown in Figure S1. Median fluorescence intensity (MFI) values were converted to MESF values using Quantum^TM^ MESF beads (Bangs Laboratories, 823, Fishers, IN, USA) conjugated to APC according to the manufacturer’s protocol and converted to MESF units using QuickCal^®^ v2.3 (Table S3).

### 2.7. CD107a Degranulation Assay

GBM43 target cells were allowed to attach in wells for at least 6 h. Then, wells were washed with 1× PBS, and effector NK cells cultured in either RPMIf or OpTmizer™ were added at E:T ratio of 2.5:1. For blockade assays, NK cells were pre-treated with 10 µg/mL anti-TIM-3 blocking antibody overnight. CD107a-APC (Biolegend, 328620) was added immediately after NK cell addition. After 1 h, GolgiStop (containing monensin, BD Biosciences, 554724) was added for the detection of CD107a. Samples were incubated for another 3 h before collection and surface staining of CD56-PECy5.5 and CD3-PECy7 for flow cytometry.

### 2.8. IFNγ Secretion Assay

GBM43 target cells were allowed to attach in wells for at least 6 h. Then, wells were washed with 1× PBS, and effector NK cells cultured in either RPMIf or OpTmizer™ were added at E:T ratio of 2.5:1. After 1 h, GolgiPlug (containing brefeldin A, BD Biosciences, 555029) was added for the detection of interferon gamma (IFNγ). Samples were incubated for another 3 h before fixation and permeabilization for intracellular staining.

### 2.9. Bioinformatics Analysis of GBM Patient Data

Glioblastoma (GBM) RNA-seq data were downloaded from TCGA [26] and patients (*n* = 156) were classified into high/low groups based on expression of *HAVCR2*, based on upper and lower quartiles, which are 50% for each high and low. Ultimately, this classification identified 78 samples with high *HAVCR2* expression and 78 samples with low HAVCR2 expression. Next, differential expression (DE) analysis was performed using edgeR [27,28] between high vs. low groups to identify statistically significant differentially expressed genes. False discovery rate was controlled using the Benjamini–Hochberg method [29] at α = 0.05. Next, a gene set enrichment analysis (GSEA) [30] was performed with the KEGG, Go-Biological Processes (GO.BP) and Immunologic collections from MSigDB. GSEA was performed using the FGSEA tool and genes were ranked by log2FC values. Additionally, a custom NK gene set was used in performing a GSEA, comprised of five genes (*NCR1*, *NCR3*, *KLRB1*, *CD160*, *PRF1*) [31] that have been known to have high expression in NK cells.

### 2.10. Statistical Analysis

All statistical analyses were performed with GraphPad PRISM 8 (GraphPad Software, San Diego, CA, USA). Data were presented as mean ± standard error of the mean (SEM). Assuming normal distribution, differences between two groups were evaluated using paired two-tailed Student’s t-test, with a *p*-value < 0.05 considered as significant.

### 2.11. Ethics Statement

Written informed consent was obtained from all subjects involved in the study. All procedures performed in studies involving human participants were approved by Purdue University’s Institutional Review Board (IRB) in August 2018 (#1804020540). All institutional safety and biosecurity procedures were adhered to.

## 3. Results

### 3.1. NK Cells Downregulate Expression of TIM-3 in Response to Cancer Targets

**Figure 1:**
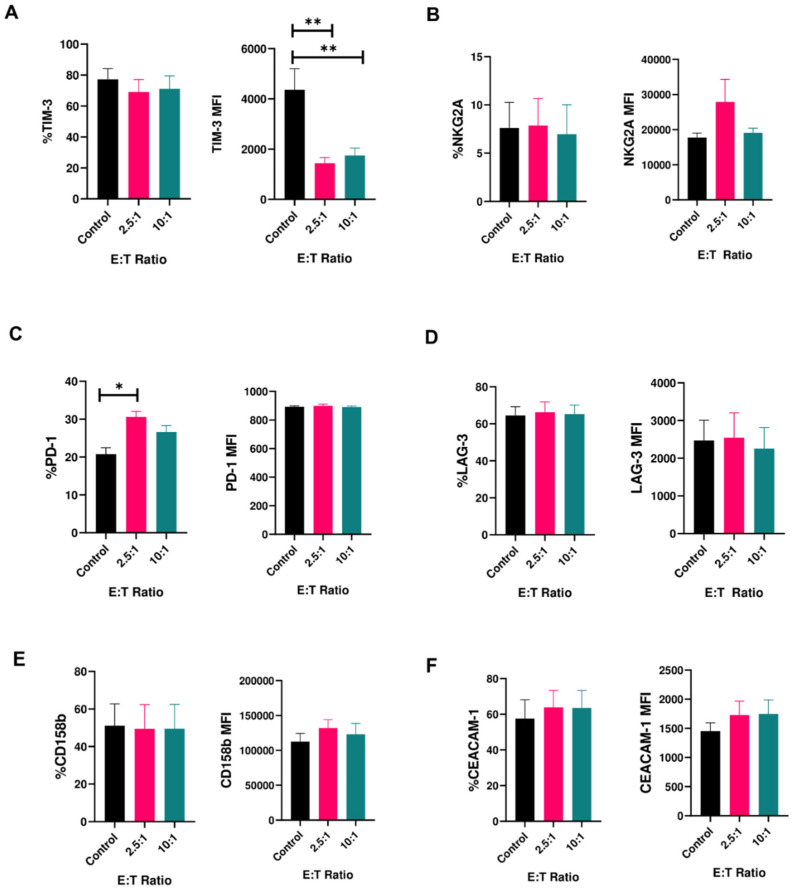
Expression of inhibitory receptors on human NK cells in response to cancer cells (mean ± SEM). Percentage (**left panels**) and median fluorescence intensity (MFI) (**right panels**) of (**A**) T-cell immunoglobulin and mucin-containing domain (TIM-3); (**B**) NGK2A; (**C**) PD-1; (**D**) LAG-3; (**E**) CD158b; (**F**) CEACAM-1 on peripheral blood-derived human NK cells upon co-culture with U87MG cells for 4 h at effector:target (E:T) ratios of 2.5:1 and 10:1 (*n* = 6–9 samples). * *p* < 0.05, ** *p* < 0.01.

We measured expression of the receptors PD-1, CEACAM-1, LAG-3, CD158b, NKG2A and TIM-3 on activated human peripheral blood-derived NK cells prior to and after exposure to cancer targets. Except TIM-3, these receptors are generally considered inhibitory to NK cell function. When exposed to glioblastoma (U87MG) cells, NK cells showed no decrease in expression of any of the receptors assayed except TIM-3 (Figure 1A–F and Figure S2; Table S1). After exposure to cancer targets, though the percentage of cells expressing TIM-3 stayed the same, the median fluorescence intensity (MFI) for TIM-3 decreased significantly, by 2926 and 2620 Intensity Units, for 2.5:1 and 10:1 effector(E): target (T) ratios, respectively (Figure 1A). This decrease was consistent across multiple donors. Among other receptors, an increase in the percentage of PD-1^+^ NK cells also occurred, but this was not accompanied by a change in MFI (Figure 1C).

**Figure 2:**
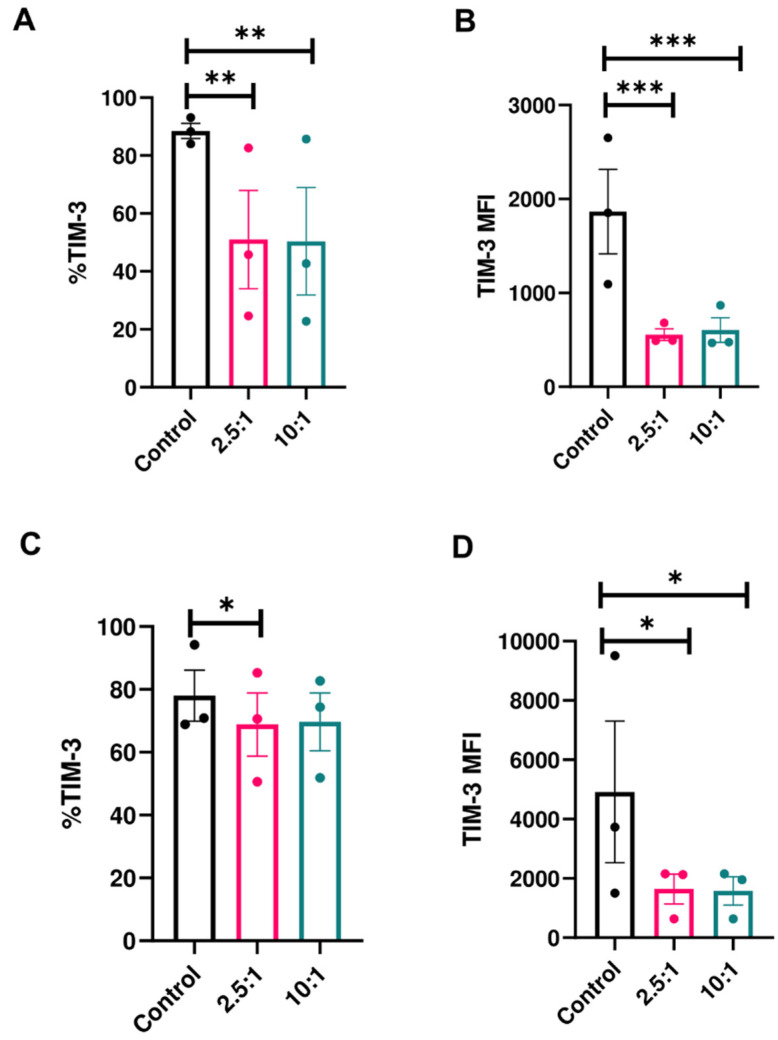
Expression of TIM-3 on NK cells in response to cancer cells (mean ± SEM). Percentage (left panels) and MFI (right panels) of TIM-3 on human peripheral blood NK cells in response to (**A**,**B**) Prostate cancer (PC3) (*n* = 3 donors) and (**C**,**D**) primary human glioblastoma (GBM43) cells (*n* = 3) after 4-h co-culture at E:T ratios of 2.5:1 and 10:1.* *p* < 0.05, ** *p* < 0.01, *** *p* < 0.001.

To establish whether the decrease in TIM-3 expression on NK cells is unique to the U87MG cancer cell line, we exposed NK cells to prostate cancer (PC3) and patient-derived glioblastoma (GBM43) cells. In both cases, we observed a decrease in expression of TIM-3 on peripheral blood NK cells after exposure to cancer cells (Figure 2A–D). TIM-3 percentage decreased in the presence of GBM43 and PC3 cells (Figure 2A,C), as did the MFI (Figure 2B,D). These observations suggested that the decrease in TIM-3 expression was specific to this receptor. When looking at trends of expression of TIM-3 on human NK cells exposed to GBM43 cells for individual donors (Figure S3A,B), we observed that while expression can be variable among different healthy donors, trends in decrease following cancer cell stimulation are consistent. Additionally, no change in activating receptor expression (DNAM-1) was observed on human NK cells in response to GBM (Figure S4).

### 3.2. Media Composition Contributes to Changes in TIM-3 Expression on Activated NK Cells

The observed decrease in TIM-3 expression on peripheral blood NK cells when exposed to cancer targets prompted us to question the conditions which lead to the induction of such a decrease. To that end, we sought to determine the role of stimulation conditions on TIM-3 expression on peripheral blood NK cells exposed to GBM. We assembled a matrix of stimulation and culture conditions to which NK cells would be exposed (Table 1). For each condition, we measured both percentage of TIM-3^+^ NK cells and the surface density of TIM-3 expression (as MFI).

**Figure 3:**
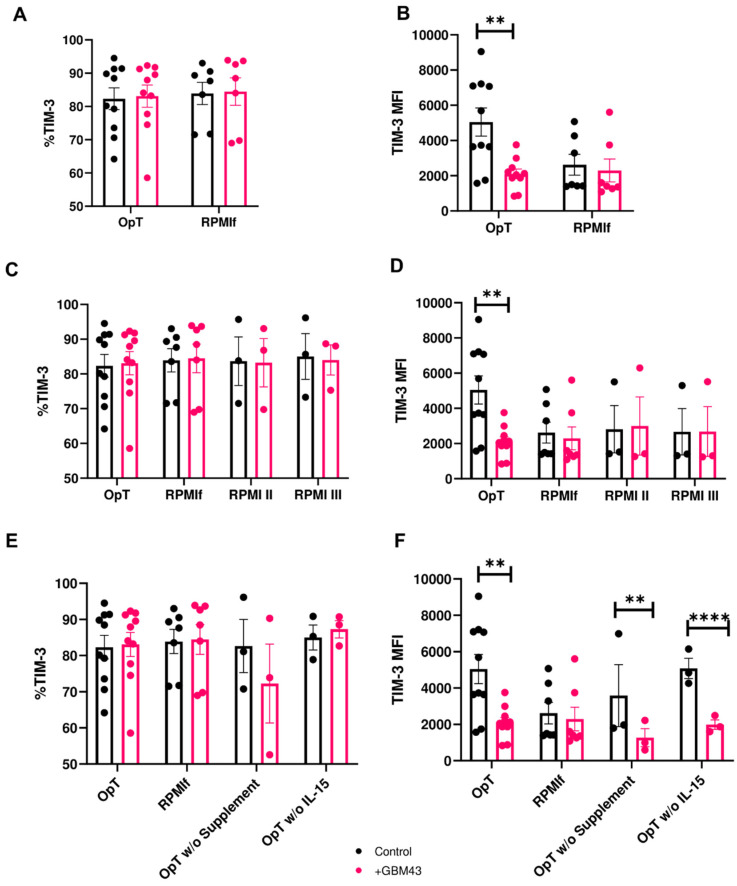
Expression of TIM-3 on NK cells in response to cancer cells under varying stimulation conditions of cytokine and supplement compositions (mean ± SEM). Percentage and MFI of TIM-3 were measured on human NK cells after 4 h co-culture with GBM43 cells at an E:T ratio of 2.5:1. (**A**,**B**) TIM-3 expression on NK cells expanded in complete NK-modified OpTmizer™ (OpT) (*n* = 10 donors) or RPMI-based expansion medium (RPMIf, *n* = 7); (**C**,**D**) TIM-3 expression on NK cells expanded in OpT, RPMIf, RPMI medium modified to contain 10 ng/mL rhIL-15 and 25 ng/mL rhIL-21 (RPMI II, *n* = 3) or RPMI medium modified to contain 10 ng/mL rhIL-15 (RPMI III, *n* = 3); (**E**,**F**) TIM-3 expression on NK cells expanded in OpTmizer™ (OpT), RPMIf, OpT medium without supplement (OpT w/o supplement, *n* = 3), or OpT without IL-15 (OpT w/o IL-15, *n* = 3). ** *p* < 0.01, **** *p* < 0.0001.

NK cells were first cultured in either complete NK-modified OpTmizer™ medium (containing IL-2, IL-15 and IL-21) or our original RPMI-based expansion medium (typically used in conjunction with K562 feeder cells, containing RPMI-1640 supplemented with 4-1BBL, IL-2 and IL-21, labeled RPMIf) and then exposed to GBM43 cells. Because previous experiments showed no significant difference in change in TIM-3 expression between various E:T ratios, we chose an E:T ratio of 2.5:1 for further experiments. Upon co-culture with GBM43 cells, NK cells cultured in OpTmizer™ medium retained consistent TIM3^+^ percentage levels (Figure 3A), but showed the same reduction in TIM-3 expression (as MFI) observed previously (Figure 3B), while cells grown in RPMIf medium did not exhibit change in expression of TIM-3 either in terms of percentage (Figure 3A) or MFI (Figure 3B), a finding that was consistent across multiple donors (Figure S5). Downregulation in NK cell TIM-3 expression upon exposure to cancer targets was also observed on resting NK cells in the absence of supplement or cytokine stimulation (Figure S6). Interestingly, NK cells activated in OpTmizer™ medium also had an upregulated starting level of TIM-3 expression compared to RPMIf-expanded NK cells (Figure 3A,B). This was true in all experimental setups, and could be an indication that OpTmizer^TM^ medium upregulates TIM-3 expression which is then tempered down to normal pre-activation levels by cancer targets. Despite this higher level of initial TIM-3 expression, its decrease on OpTmizer™-stimulated NK cells was consistent and significant.

### 3.3. IL-15 Alone Is not Responsible for Downregulation in TIM-3 Expression

Because the downregulation appeared to be driven by contact with cancer cells in potential synergy with a combination of factors which the NK cells had been exposed to during activation and expansion, we were interested in investigating further to what extent medium composition might be contributing to changes in TIM-3 expression. First, we investigated the contribution of individual cytokines or cytokine combinations. TIM-3 expression was previously reported to be induced by specific cytokines, namely IL-2, IL-12, IL-15, IL-18 and IFNα [3], and we were interested in finding out if the same applied to in vitro-expanded human peripheral blood NK cells. To that end, we cultured NK cells in modified media by varying individual components to establish their contribution to the observed behavior. With OpTmizer™ as a control, we prepared three variants of NK expansion media: the original NK expansion medium (RPMIf), NK expansion medium with a cytokine composition that matched that of OpTmizer™ medium (RPMI II; Table 1)—achieved by the addition of IL-15 and a reduction in IL-21 concentration—and original RPMIf medium with the addition of IL-15 (RPMI III; Table 1). When NK cells cultured in these various media were exposed to GBM cells, the lack of observed change in percentage of TIM-3^+^ NK cells (Figure 3C) accompanied by a downregulation in TIM-3 expression of 2939.9 Intensity Units was only seen on NK cells cultured in OpTmizer™ medium (Figure 3D), while all RPMI variants showed consistent TIM-3 expression on NK cells after exposure to cancer targets. NK cells activated in OpTmizer™ medium again showed an initial upregulation in TIM-3 MFI levels prior to the cancer-induced downregulation. This suggested that the decrease in TIM-3 expression was not driven by IL-15 alone or the composition of cytokines present in OpTmizer™ medium. This did not rule out, however, the contribution of IL-15 or other cytokines in combination with additional stimulatory factors. Although a minor (2.2%) increase in TIM-3^+^ NK cells in RPMI III medium was recorded, this was not accompanied by any change in TIM-3 MFI levels.

**Figure 4:**
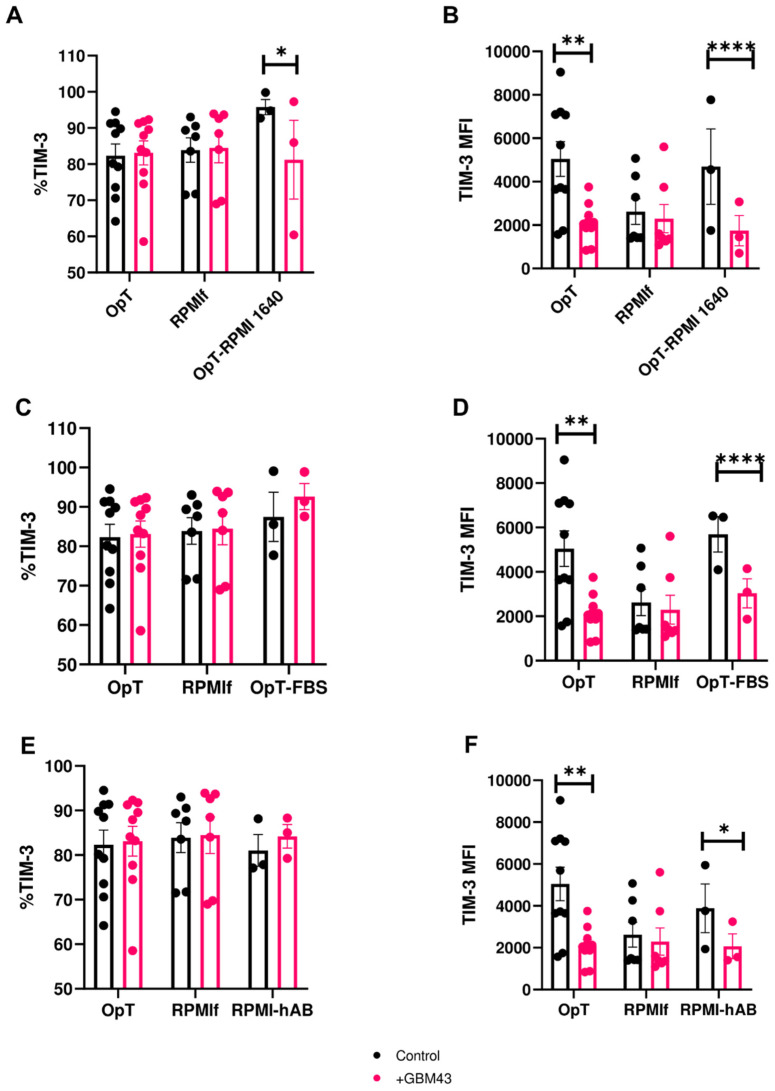
Expression of TIM-3 on NK cells in response to cancer cells under varying media and serum compositions (mean ± SEM). Percentage and MFI of TIM-3 were measured on human NK cells after 4 h co-culture with GBM43 cells at an E:T ratio of 2.5:1 (*n* = 3–10 donors). (**A**,**B**) TIM-3 expression on NK cells expanded in OpT (*n* = 10), RPMIf (*n* = 7) or OpT with RPMI-1640 as the basal medium component (OpT-RPMI 1640, *n* = 3); (**C**,**D**) TIM-3 expression on NK cells expanded in OpT, RPMIf or OpT with fetal bovine serum FBS instead of human AB serum (*n* = 3); (**E**,**F**) TIM-3 expression on NK cells expanded in OpT, RPMIf, or RPMIf with human AB serum instead of FBS (*n* = 3). * *p* < 0.05, ** *p* < 0.01, **** *p* < 0.0001.

In order to infer which component of the OpTmizer™ recipe played a more direct role in contributing to the changes in TIM-3 expression on NK cells, we modified OpTmizer™ medium by removing either the recommended cytokine supplement (OpTmizer™ (OpT) w/o supplements) or removing IL-15 alone (OpT w/o IL-15) and then exposed NK cells activated in these media to GBM cells as before. Under these conditions, the percentage of TIM-3^+^ NK cells remained largely unchanged across donors (Figure 3E). However, in both OpTmizer™ variants, TIM-3 MFI decreased after exposure of NK cells to cancer cells, by 2871.3 and 3093.7 Intensity Units for OpTmizer™ medium without supplements and OpTmizer™ medium without IL-15, respectively (Figure 3F). These trends were consistent for multiple donors tested (Figure S5). This experiment indicated that neither IL-15 alone, nor a single component of the OpTmizer™ supplement was responsible for the downregulation in TIM-3 expression on NK cells. We postulated that a basal media component could be acting alone or in concert with other stimulation factors as the culprit. This assumption was tested by activating NK cells in OpTmizer™ medium modified by replacing its basal component with RPMI-1640 (OpT-RPMI 1640) and then exposing them to GBM cells as before. These conditions again induced downregulation in TIM-3 expression on NK cells after cancer cell contact, both in percentage (Figure 4A) and MFI (Figure 4B), from upregulated basal levels, a behavior that was not reproduced on NK cells cultured in either complete OpTmizer™ or RPMIf media (Figure 4A,B). Based on these observations, basal media was not considered as the driver of the observed changes in TIM-3 expression.

### 3.4. Human Serum Contributes to Changes in TIM-3 Expression on NK Cells

We next investigated the role of human serum, which had been added, as human AB serum, at a concentration of 5% to each OpTmizer™ recipe. To do so, we first prepared a variant of OpTmizer™ medium by replacing human AB serum with FBS (OpT-FBS) at the same concentration (5%). Exposure of human NK cells activated in FBS-containing OpTmizer™ (OpT-FBS) to GBM cells led to a small (7.92%) increase in TIM-3^+^ NK cells (Figure 4C), but a decrease in TIM-3 MFI expression following basal upregulation as observed before (Figure 4D).

However, when NK cells were activated in a variant of RPMI-based feeder medium wherein FBS was replaced with human AB serum (RPMI-hAB), we observed, alongside a consistent percentage of TIM-3^+^ NK cells (Figure 4E), a significant decrease in TIM-3 MFI levels of 1816 Intensity Units on human NK cells after exposure to cancer cells (Figure 4F). As before, these trends were consistent for each of multiple donors (Figure S7). Similar to prior observations with OpTmizer™ media, NK cells in RPMI-hAB had upregulated basal levels of TIM-3. This matched the decrease in TIM-3 expression observed earlier on NK cells activated in complete OpTmizer™ medium. Because such a decrease was also present when FBS replaced human AB serum in the original OpTmizer™ recipe, it appears that human AB serum alone is not sufficient to induce changes in TIM-3 expression, and it is likely to be acting in combination with another agent which NK cells respond to. It should be noted that the magnitude of decrease in TIM-3 expression was over 1.5 × greater for OpTmizer™ (~2958 Intensity Units) than for RPMI-hAB (1816 Intensity Units) medium. Moreover, data in Figure 4F indicate that human AB serum is not the only driver of TIM-3 expression changes, but media-specific components unique to OpTmizer™, likely in combination with one another, are at play. Because cytokines, particularly IL-2 and IL-15, had been reported as contributing to the modulation of TIM-3 expression on NK cells [3], their synergism with either human AB serum or FBS is possibly driving changes in TIM-3 expression. Collectively, our data indicate that downregulation of TIM-3 is likely to be primarily driven by cancer cells, with stimulation conditions contributing to this change by upregulating basal levels of TIM-3.

### 3.5. Downregulation in TIM-3 Expression Correlates to Impaired NK Cell Cytotoxicity

**Figure 5:**
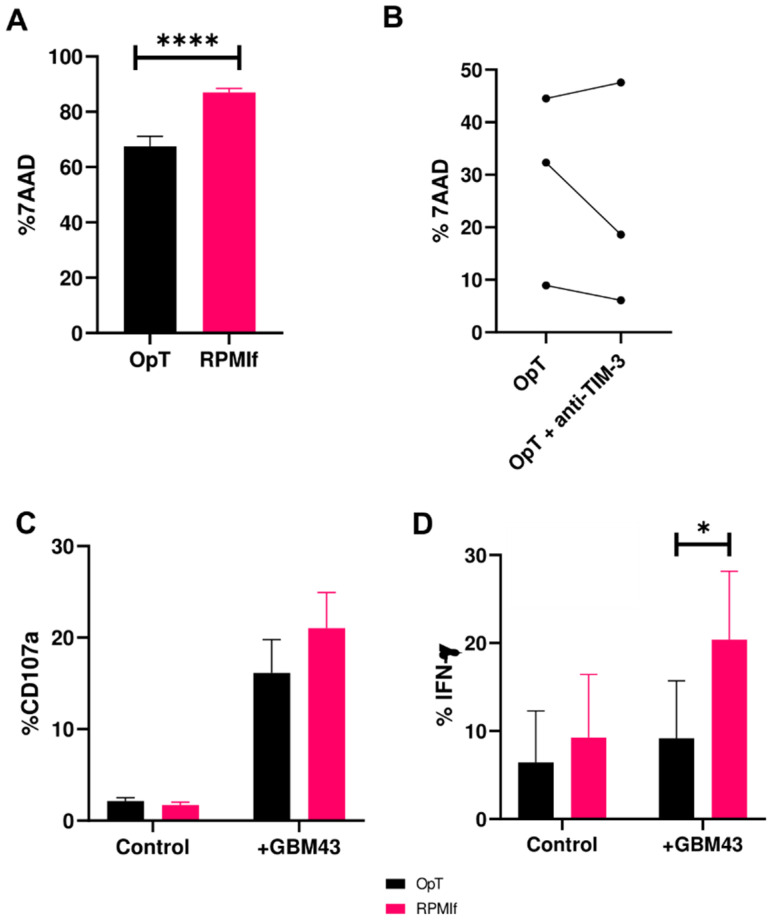
Functional potential of human NK cells against GBM43 cells under varying stimulation conditions (mean ± SEM). Cytotoxicity of human NK cells after 4 h co-culture with GBM43 cells at an E:T ratio of 2.5:1 following stimulation in either (**A**) OpT or RPMIf medium (*n* = 3 donors); and (**B**) following TIM-3 blockade stratified for three individual donors (*n* = 3); (**C**) CD107a and (**D**) IFN-γ production (*n* = 3) by NK cells stimulated in OpT or RPMIf in response to GBM43 cells. * *p* < 0.05 and **** p < 0.0001.

Because TIM-3, largely thought to be an exhaustion marker on NK cells, had been described to possess stimulatory roles, we sought to determine whether the observed downregulation of its expression on peripheral blood NK cells correlated to altered function. To study this, we challenged NK cells, activated as before, to kill patient-derived primary glioblastoma cells (GBM43). Stimulation conditions that led to the downregulation of TIM-3 expression correlated to a lower cytotoxicity against GBM43 cells (Figure 5A). Generally, NK cells cultured in OpTmizer™ had a poorer ability to kill GBM43 cells than NK cells expanded in RPMIf medium, which had also shown no loss in TIM-3 expression. These trends in killing ability were consistent for each of multiple donors tested (Figure S8), and were also observed with resting NK cells, which had been cultured in the absence of medium supplement or cytokines (Figure S9). This diminished cytotoxicity was present despite TIM-3 expression following co-culture with cancer cells being almost equivalent between OpTmizer^TM^ and RPMIf-cultured NK cells, suggesting that activation in OpTmixer^TM^ might be inducing impairment in NK cell lytic ability.

There was no significant change in NK-mediated killing of target cells following antibody blockade of TIM-3 (Figure 5B). NK cells, activated in either OpTmizer™ or RPMIf medium showed no observable change in their ability to kill GBM43 cells following addition of anti-TIM-3 antibody (clone F38 2E2). This was consistent across separate donors despite their difference in NK cell killing capacity. Similar results were obtained for TIM-3 blockade in RPMIf medium (Figure S10).

Consistent with the impaired cytotoxicity of NK cells activated in OpTmizer™ medium, the ability of these cells to produce cytokines was also impaired following stimulation by cancer cells. OpTmizer™-activated NK cells were observed to be inferior producers of IFN-γ compared to NK cells activated in RPMIf (Figure 5D). Unlike changes in the overall killing of target cells and IFN-γ production observed between the two different in vitro stimulation conditions, degranulation of NK cells remained unchanged between OpTmizer™ and RPMIf (Figure 5C). Specifically, expression of CD107a on NK cells was consistent among both conditions (Figure 5C) and showed modest change in the presence of an anti-TIM-3 antibody (Figure S11). The lack of significant change in degranulation could be a result of the pre-activation of NK cells by cytokines, which have been shown to have the ability to induce degranulation of NK cells. Moreover, the mechanisms by which NK cells induce killing of target cells are not only dependent on CD107a expression, indicating that other factors driving killing potential are at play. These findings suggest that specific stimulation conditions—in our case, activation in OpTmizer^TM^—might be driving a defect in NK cell cytotoxicity. Furthermore, media stimulation can work in synergy with cancer targets to induce changes in TIM-3 expression, wherein loss of TIM-3 upon co-culture with cancer cells synergizes with cell-intrinsic effects of media stimulation to result in the concomitant impairment in their cytotoxic potential. In other words, lower TIM-3 appears to correlate and possibly contribute to lower cytotoxicity, but does not directly cause it.

### 3.6. GBM Patient Data Show Correlation between TIM-3 Expression and NK Cell Function

**Figure 6:**
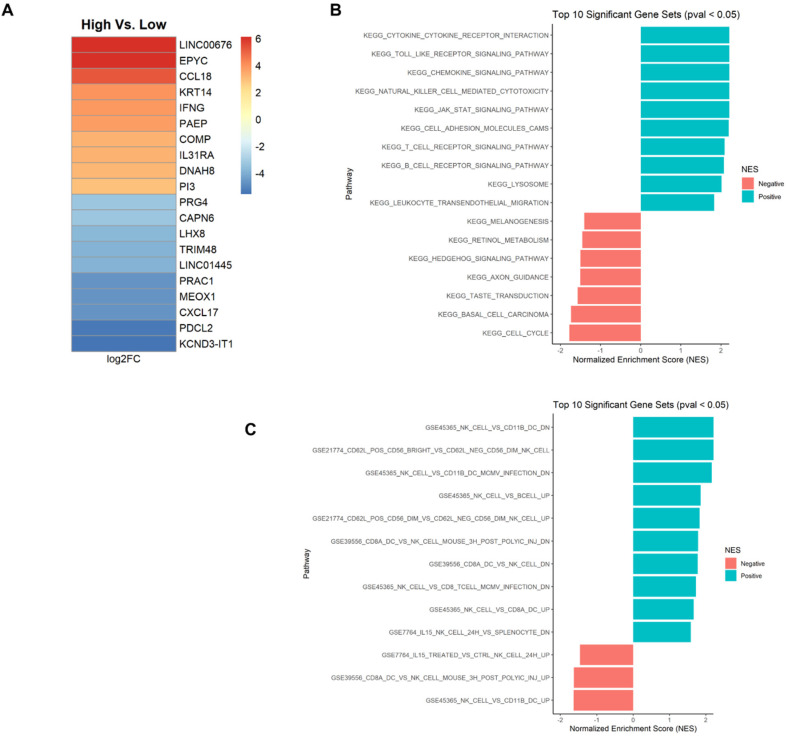
Bioinformatics analysis of GBM patient data (TCGA) stratified for *HAVCR2* (TIM-3) expression and correlation to NK cell function. (**A**) Heatmap of top 10 up- and down-regulated DE genes in *HAVCR2*^+^ GBM patients show upregulated *IFNG*; (**B**) GSEA analysis with KEGG collection from the Molecular Signatures Database (MSigDB) shows upregulation of NK cytotoxicity and JAK/STAT signaling pathways in *HAVCR2*^+^ samples; (**C**) NK cell-related gene sets from KEGG, GO.Biological Processes and Immunologic collections show enrichment in CD62L^+^CD56^dim^ NK cell presence on TIM-3^+^ GBM.

Bioinformatics analysis of gene expression from GBM patient data (TCGA) revealed a significant positive correlation between the custom five gene NK signatures and TIM-3 (*HAVCR2*) expression in GBM (*p*-value < 0.25; Table S2), correlating a high expression of the TIM-3 gene with higher NK presence. We performed an analysis of the top 10 up- and down-regulated genes in GBM patient samples that we had stratified and identified as overexpressing TIM-3 (Figure 6). A volcano plot (Figure S12) generated from DE analysis shows differentially-expressed genes in relation to *HAVCR2* in the analyzed GBM patient datasets. We found that *IFNG* was among the most highly upregulated genes (Figure 6A, logFC = 3.91). This observation correlated with the substantial increase in expression of IFN-γ we observed upon co-culture of NK and GBM cells (Figure 5D). Interestingly, among the most highly downregulated genes in TIM-3^+^ GBM samples included *MEOX1* (mesenchyme homeobox 1), *CAPN6* (calpain 6), *PRAC1* (prostate cancer susceptibility candidate protein 1) and *CXCL17*. These genes carry out various distinct roles, but have also been associated with the capacity to exert pro-tumorigenic functions in a number of cancers. 

From the GSEA analysis, the top significant (*p*-value < 0.05) gene sets from the KEGG (Figure 6B), GO.BP (Figure S13A) and Immunologic (Figure S13B) collections are shown. KEGG analysis of GBM patient samples with high TIM-3 expression showed a positive correlation with pathways associated with NK cytotoxicity, Janus kinase/signal transducer and activator of transcription protein (JAK/STAT) signaling and chemokine signaling (Figure 6B). Gene sets related to NK cells were identified and filtered out from enrichment results of all three collections, the plotted with significance cutoff *p*-value < 0.05 (Figure 6C). This revealed a significant pathway associated with the CD62L^+^CD56^dim^ NK phenotype, described as defining intermediate maturation, a high capacity to produce IFN-γ and competent cytotoxic ability.

## 4. Discussion

TIM-3 is a somewhat peculiar receptor: though widely expressed on NK cells, its expression can be modulated ex vivo or in vitro based on the specific NK stimulation program. In vitro, studies have shown that TIM-3 expression could be induced on human NK cells by the cytokines IL-2, IL-12, IL-15, IL-18 and IFNα, as well as following FcR interactions with NK cells [3,13,25]. This hasn’t been mirrored in vivo, however, where elevated expression of TIM-3 on NK cells from the peripheral blood of cancer patients has been correlated with poor prognosis and a dysfunctional NK cell state in a variety of cancers, such as lung adenocarcinoma [32] and melanoma [6].

TIM-3 has been described as both inhibitory and activating to NK cells. Its inhibitory role was described by Ndhlovu and colleagues, who showed that when TIM-3 was crosslinked with anti-TIM-3 antibodies, it caused suppression of NK cell killing otherwise mediated by a variety of NK activating antibodies, including NKG2D and CD16 [3]. Gleason and colleagues, on the other hand, reported that TIM-3 was able to enhance IFN-γ production from human NK cells following binding to one of its ligands, galectin-9 [4], prompting them to describe TIM-3 as an activating receptor. These seemingly conflicting results have been rationalized by the notion that TIM-3 might exhibit both stimulus- and environment-specific functions. So and colleagues argued the former by showing that depending on activation stimulus, TIM-3 expression of NK cells correlates to different rates of IFN-γ and CD107a production [25]. Our data further fuel this point; however they also hint at environment-specific activation triggers to be a factor.

Results on the role of TIM-3 in appear more comprehensive within the context of T cells. TIM-3 was established as a negative regulator of T cell anti-tumor responses due to its association with an exhausted T cell state marked by the progressive loss of these cells’ ability to express IFN-γ, TNF-α and IL-2 [33,34,35]. TIM-3^+^ CD8^+^ T cells were also shown to exhibit impaired signal transducer and activator of transcription 5 (STAT5) and p38 signaling [36]. However, conflicting evidence has suggested that TIM-3 may have a stimulatory effect on CD8^+^ T cells in acute infection [37]. Interestingly, Isshiki and colleagues [38] reported that anti-TIM-3 therapy was associated with severe lung inflammation and fibrosis, due to elevated accumulation of TGF-β1 and apoptotic cells in the lungs following anti-TIM-3 treatment. Galectin-9, the most commonly studied TIM-3 ligand [39], triggers TIM-3-mediated inhibition of immune cell function and results in T cell apoptosis and loss of immune protection [40]. In the absence of galectin-9 binding, TIM-3 can interact with human leukocyte antigen (HLA)-B-associated transcript 3 (Bat3), which was shown to protect T cells from TIM-3-induced immune inhibition [41].

Though it was described as both inhibitory and activating, the exact role of TIM-3 on NK cells is not fully clear. Our study supports the notion that TIM-3 expression is dependent on the specific stimulus used to modulate its expression, and that such expression does not define an exclusively stimulatory or inhibitory role on NK cells. We observed a phenomenon wherein expression of TIM-3 on NK cells is downregulated when these cells, pre-activated in vitro under certain conditions, are exposed to active cancer targets. It is likely, our data suggest, that the downregulation is a correction of TIM-3 to baseline levels following cancer cell contact and after initial upregulation in specific media. Cancer cells investigated in our study—GBM43 and U87MG—express TIM-3 ligands, including galectin-9 and CEACAM-1, with both dominating in percentage on GBM43 cells and in MFI on U87MG cells (Figure S14). Downregulation of TIM-3 expression was specific to TIM-3, rather than other inhibitory NK cell receptors (Figure 1) and occurred in the presence of multiple cancer cell types to varying extents (Figure 2). Interestingly, PD-1 was the only other receptor among those tested to have recorded a change, albeit in the opposite direction: the proportion of PD-1^+^ NK cells increased following stimulation by cancer cells. This pattern is the opposite of what had been observed in tumor infiltrating T-cells, where both PD-1 and TIM-3 tend to increase due to T-cell exhaustion [42], and therefore warrants further investigation. In terms of TIM-3 expression, specifically, the net negative change stemmed from an initial upregulation in TIM-3 expression when these cells were activated in OpTmizer™ medium, followed by downregulation upon contact of NK cells and cancer targets. A combination of stimulation conditions that include the use of serum (specifically human AB serum), cytokine mixtures (Table 2), and possibly effector molecules secreted by target cells appeared to contribute to this phenomenon. Out of eight different media recipes tested, all but two resulted in TIM-3 downregulation (Table 2). Some of these observations confirm earlier findings that TIM-3 is an inducible receptor, able to be modulated by various activation stimuli. Because RPMIf NK cells did not exhibit similar TIM-3 expression change when exposed to the same cancer target, it could be that these two sets of expanded NK cells are of two distinct activation statuses, and that TIM-3 expression was induced to respond to the degree of activation experienced by NK cells through external stimuli.

In addition, hints that TIM-3 may have a stimulatory role on NK cells emerged from our study. A decrease in TIM-3 expression correlated to impaired cytotoxicity—NK cells whose TIM-3 expression was downregulated killed significantly less than NK cells whose TIM-3 expression levels remained statistically unchanged, and the former also produced significantly less IFN-γ (Figure 5D). This appeared to be driven by an intrinsic defect in NK cytotoxicity that activation in OpTmizer^TM^ medium appeared to induce, not due to TIM-3 itself. This downregulation, and the associated reduction in cytotoxicity, appeared to be linked to upregulated basal levels of TIM-3 since attempts to preserve the heightened TIM-3 expression of OpTmizer™ NK cells with antibodies did not show changes in cytotoxicity compared to samples without antibodies. It could be possible that TIM-3 acts, in fact, as a marker of activation status—and potentially exhaustion—of NK cells ex vivo or in vitro, the expression of which correlates to the cells’ cytotoxicity. TIM-3 blockade not rescuing the impaired cytotoxicity further implies that TIM-3’s role under the conditions studied here may be less that of functional modulation and more of a status marker. In summary, TIM-3 expression appeared to correlate to NK functional status, but not drive it.

The mechanisms by which TIM-3 expression is induced are not entirely known, nor are the effects of cancer cells on TIM-3 expression levels. Recently, the induction of TIM-3 expression on NK cells was reported to be induced by MHC class I-deficient tumor cells that come into contact with NK cells [43]. Our study shows that in vitro, the opposite may be true—cancer cells have the ability to induce a decrease in TIM-3 expression on expanded, activated NK cells. Bioinformatics analysis of GBM patient data revealed a strong positive correlation between TIM-3 expression and NK cell presence, and confirmed a role for *IFNG* in these patients, possible via JAK/STAT activation, confirming some of our in vitro data. Transcriptional data point to a nuanced role of TIM-3 in GBM. The association between high TIM-3 expression and the downregulation of genes including *MEOX1, CAPN6, PRAC1* and *CXCL17* in GBM suggests the role of this receptor may not be fully inhibitory, owing to the pro-tumorigenic roles that have been described for these genes [44,45,46,47,48]. TIM-3, instead, appears to be involved in a varied set of immunological responses in GBM.

TIM-3′s stimulatory role, at least as it pertains to T cells, was linked to a signaling cascade induced by tyrosine phosphorylation of the receptor’s cytoplasmic tail [49]. This triggers activation of T-cell receptor (TCR)- and CD28-dependent signaling pathways resulting in nuclear factor of activated T-cells/activator protein 1 NFAT/AP-1 and nuclear factor kappa-light-chain-enhancer of activated B cells (NF-κB)-dependent transcription, and IL-2 production. Such activation is considered finite; however, upon prolonged stimulation, TIM-3-mediated overactivation of TCR/CD3 signaling is thought to favor an exhausted phenotype at the expense of memory T cell development, triggering the “inhibitory” role of TIM-3 [50]. We may be seeing hints of that here: NK cells which upregulate TIM-3 can respond to cancer targets by assuming an altered, more “exhausted,” activation status marked by lower TIM-3 expression and a lower cytotoxicity under certain activation conditions. Such an “activation limiting” function of TIM-3 was suggested previously [51]. In our case, overexpression of TIM-3 in OpTmizer™ medium follows NK activation and results in the eventual need for correction, resulting in downregulation to preserve NK cell function. However, such compensation might not have been sufficient to reverse the functional consequence of the prior TIM-3 overexpression. Though upregulation of TIM-3 is likely due to media stimulation, downregulation is likely driven primarily by cancer cells. In other words, our data point to TIM-3 correlating to cytotoxicity of activated NK cells, but not driving it.

In summary, our data confirm earlier findings about TIM-3 being an inducible marker on NK cells, the expression of which depends on the stimulus used to induce it. However, we also highlight a previously unreported role of TIM-3 as a marker of NK cell activation status which can result in its downregulation, triggered by interaction with cancer cells in synergy with certain ex vivo stimulation conditions, and which is associated with cytotoxicity impairment, ultimately correlating TIM-3 expression with NK cell function.

## 5. Conclusions

TIM-3 has been associated with both inhibitory and activating functions on NK cells, though its exact contribution to NK activation has so far remained elusive. Our study indicated that TIM-3 is an inducible marker that correlates to different degrees of NK cell activation when exposed to external stimuli, where the highest effect appeared to come from a combination of cytokines, serum and cancer targets. In particular, it is the net expression of TIM-3 that correlated to NK cytotoxicity, specifically IFN-γ production. Downregulated TIM-3 expression was associated with lower NK cell effector activities. We speculate that TIM-3 is an activation limiter on NK cells, where there exists a threshold expression level required for NK cytotoxicity but, once exceeded, the overexpression could be inhibitory to NK effector functions.

## Figures and Tables

**Table 1:** Experimental compositions of natural killer (NK) cell culture media.

Name	Composition
RPMI II	RPMI 1640 + 10% FBS + 1%Pen/strep + 50 ng/mL 4-1BBL + 10 ng/mL rhIL-15 + 500 IU/mL rhIL-2 + 25 ng/mL rhIL-21
RPMI III	RPMI 1640 + 10% FBS + 1%Pen/strep + 50 ng/mL 4-1BBL + 10 ng/mL rhIL-15 + 500 IU/mL rhIL-2 + 50 ng/mL rhIL-21
OpT without (w/o) Supplements	OpTmizer Basal Medium + 0% OpTmizer supplement + 5% hAB serum + 1% Pen/strep + 0.2 mM l-glutamine + 10 ng/mL rhIL-15 + 500 IU/mL rhIL-2 + and 25 ng/mL rhIL-21
OpT w/o IL-15	OpTmizer Basal Medium + 2.6% OpTmizer supplement + 5% hAB serum + 1% Pen/strep + 0.2 mM l-glutamine + 0 ng/mL rhIL-15 + 500 IU/mL rhIL-2 + and 25 ng/mL rhIL-21
OpT-RPMI 1640	RPMI 1640 + 2.6% OpTmizer supplement + 5% hAB serum + 1% Pen/strep + 0.2 mM l-glutamine + 10 ng/mL rhIL-15 + 500 IU/mL rhIL-2 + and 25 ng/mL rhIL-21
OpT-FBS	OpTmizer Basal Medium + 2.6% OpTmizer supplement + 5% FBS + 1% Pen/strep + 0.2 mM l-glutamine + 10 ng/mL rhIL-15 + 500 IU/mL rhIL-2 + and 25 ng/mL rhIL-21
RPMI-hAB	RPMI 1640 + 10% hAB serum + 1%Pen/strep + 50 ng/mL 4-1BBL + 500 IU/mL rhIL-2 + 50 ng/mL rhIL-21

Deviations from the original RPMIf or OpTmizer™ recipes are underlined.

**Table 2:** Media compositions and their effect on the downregulation of TIM-3 on human NK cells in the presence of cancer cells across eight different media recipes. ‘+’ indicates the media component present in the recipe; ‘YES’ indicates that the recipe resulted in TIM-3 downregulation; ‘NO’ indicates that the media recipe did not result in TIM-3 downregulation.

Medium Component	I	II	III	IV	V	VI	VII	VIII
RPMI		+	+			+		+
OpT Basal	+			+	+		+	
OpT Supp	+				+	+	+	
AB Serum	+			+	+	+		+
FBS		+	+				+	
IL-2	+	+	+	+	+	+	+	+
IL-15	+		+	+		+	+	
IL-21	+	+	+	+	+	+	+	+
TIM-3 MFI decrease	YES	NO	NO	YES	YES	YES	YES	YES

RPMI: RPMI-1640; OpT Basal: OpTmizer™ basal medium; OpT Supp: OpTmizer™ supplement medium; AB Serum: Human AB Serum; FBS: fetal bovine serum.

## Supplementary Materials

The following are available online at https://www.mdpi.com/2072-6694/12/9/2417/s1, Figure S1: Gating strategy for stratification of TIM-3^+^ NK cells by flow cytometry, Figure S2: Visual representation of ΔMFI for NK cell inhibitory receptors upon co-culture with U87MG cells, Figure S3: Individual donor trends in expression of TIM-3 on NK cells in response to cancer cells (mean ± SEM), Figure S4: DNAM-1 expression on human NK cells in response to GBM cells, Figure S5: TIM-3 expression on human NK cells by individual donor, Figure S6: TIM-3 expression on resting human NK cells in response to GBM cells, Figure S7: TIM-3 expression on NK cells by individual donor, Figure S8: Cytotoxicity of NK cells against GBM43 cells in different media for individual donors, Figure S9: Effect of blockade of TIM-3 on the cytotoxicity of resting NK cells against GBM43 cells, Figure S10: Effect of blockade of TIM-3 on the cytotoxicity of RPMIf-expanded NK cells against GBM43 cells for individual donors, Figure S11: Effect of blockade of TIM-3 on the degranulation capacity of OpTmizer^TM^ NK cells against GBM43 cells, Figure S12: Volcano plot showing differentially expressed genes in HAVCR2^+^ GBM patient datasets, Figure S13: GSEA analysis of top up- and down-regulated genes in HAVCR2^+^ GBM patient samples (TCGA), Figure S14: TIM-3 ligand expression on cancer cells, Figure S15: Flow cytometry dot plots for TIM-3 expression on human NK cells, Table S1: ΔMFI for NK cell inhibitory receptors upon co-culture with U87MG cells, Table S2: Correlation of TIM-3 (HAVCR2) expression and NK cell presence in GBM based on bioinformatics analysis of TCGA patient data, Table S3: MESF values for MFI conditions recorded in the manuscript representing TIM-3 expression on NK cells.
