# Supplementary material for: TIM-3 Expression Is Downregulated on Human NK Cells in Response to Cancer Targets in Synergy with Activation
Source: Cancers (Basel). 2020 Aug 26;12(9):2417. doi: 10.3390/cancers12092417 (PMC7565804; doi:10.3390/cancers12092417)
Supplement: Supplementary file 1 [file cancers-12-02417-s001.pdf]

cancers

MDPI

Article

TIM-3 Expression is Downregulated on Human NK Cells in Response to Cancer Targets in Synergy with Activation

Tram N. Dao, Sagar Utturkar, Nadia Atallah Lanman and Sandro Matosevic

Supplementary Materials:

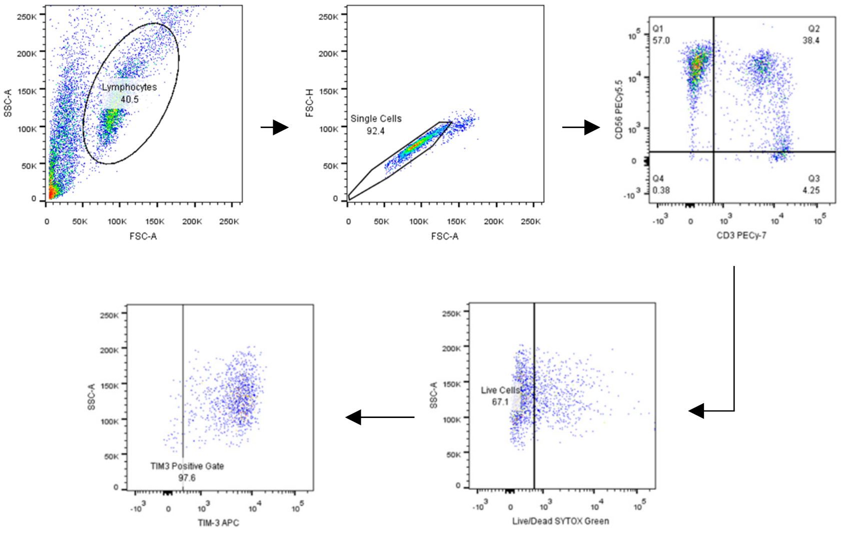

Figure S1: Gating strategy for stratification of TIM-3⁺ NK cells by flow cytometry.

Table S1. ΔMFI for NK cell inhibitory receptors upon co-culture with U87MG cells.

Receptors	$MFI_{control}$	$MFI_{2.5}$	MFI_{10}	$\Delta MFI_{2.5}$	ΔMFI_{10}
PD-1	893.16667	899.3333	891	6.166667	-2.16667
NKG2A	17796.667	27916.83	19110.33	10120.17	1313.667
TIM-3	4369.1111	1442.889	1748.889	-2926.22	-2620.22
LAG-3	2470.5	2545.167	2254	74.66667	-216.5
CD158b	112621.33	132151.8	123002	19530.5	10380.67
CEACAM-1	1450.3333	1729.667	1749	279.3333	298.6667

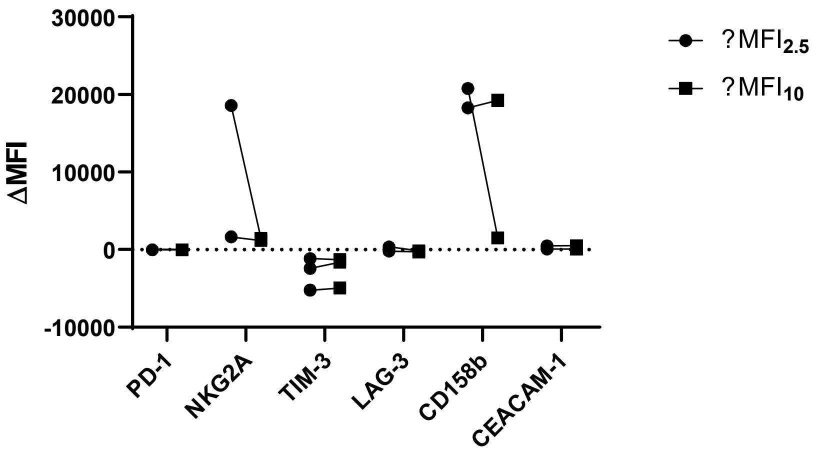

Figure S2. Visual representation of ΔMFI for NK cell inhibitory receptors upon co-culture with U87MG cells. ΔMFI was calculated by subtracting from the control MFI (NK only) (n = 6–9 independent samples).

(A)

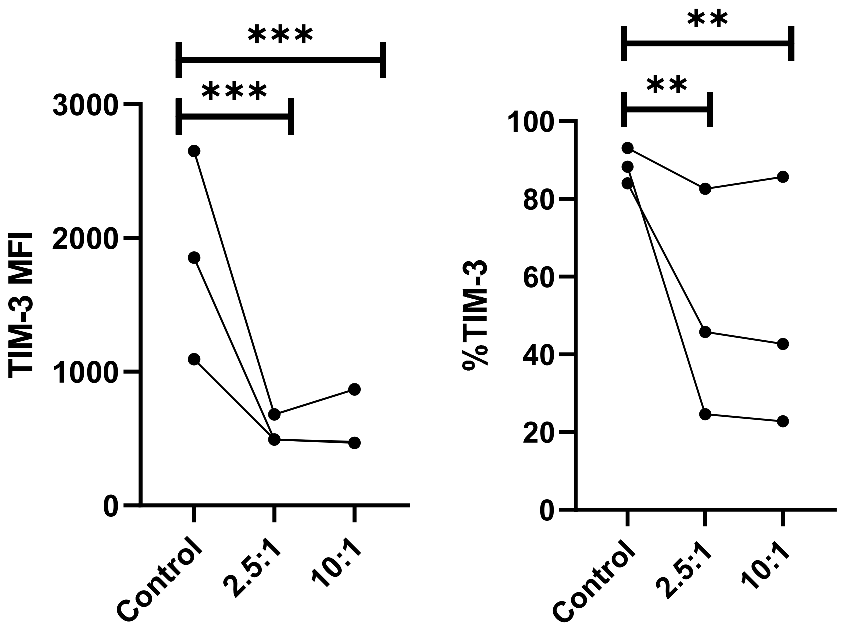

(B)

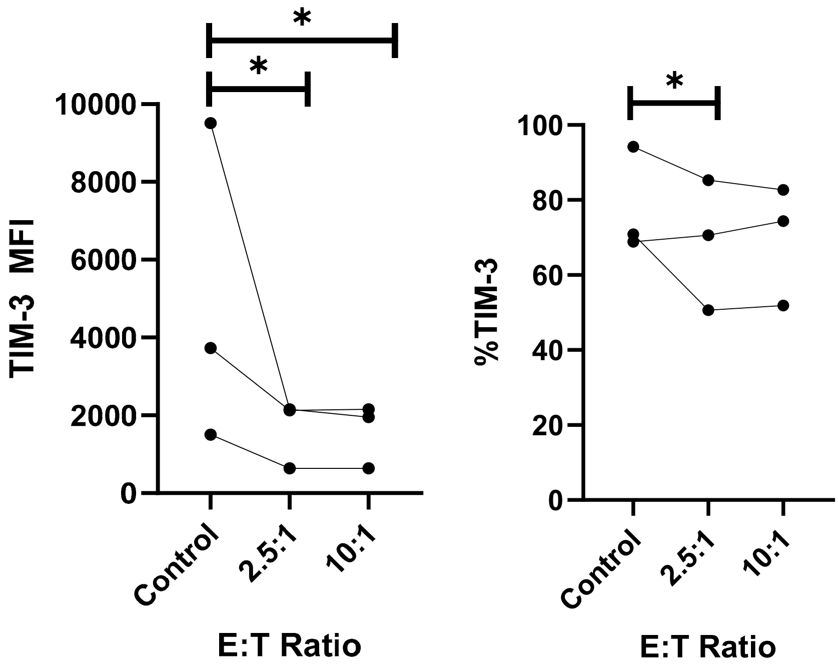

Figure S3. Individual donor trends in expression of TIM-3 on NK cells in response to cancer cells (mean ± SEM). Percentage (*left panels*) and MFI (*right panels*) of TIM-3 on human peripheral blood NK cells in response to **(A)** Prostate cancer (PC3) (n = 3 donors) and **(B)** primary human glioblastoma (GBM43) cells (n = 3) after 4-hour co-culture at E:T ratios of 2.5:1 and 10:1. **p < 0.05, **p < 0.01, ***p < 0.001.*

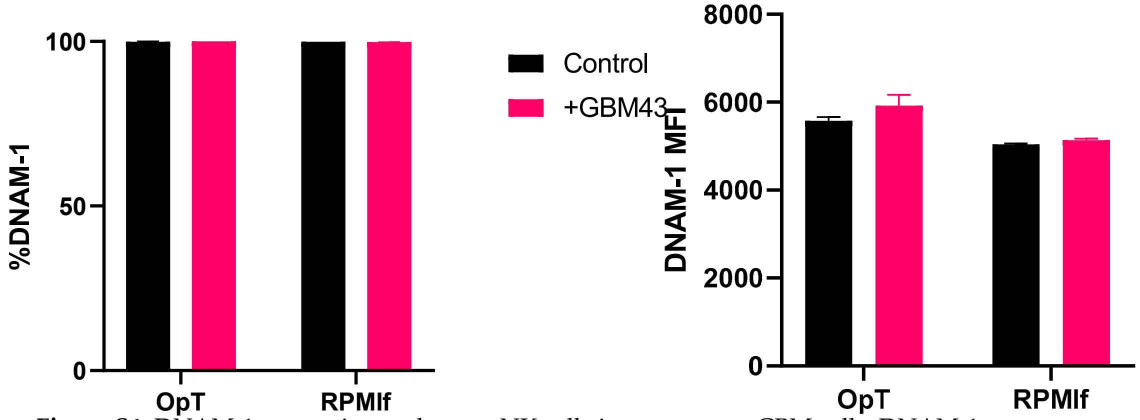

Figure S4. DNAM-1 expression on human NK cells in response to GBM cells. DNAM-1 percentage (left) and MFI (right) on NK cells upon co-culture with GBM43 cells at E:T 2.5:1 (n = 3 independent samples). No change in DNAM-1 expression was observed. All stimulation conditions were consistent and as described in Materials and Methods.

(A)

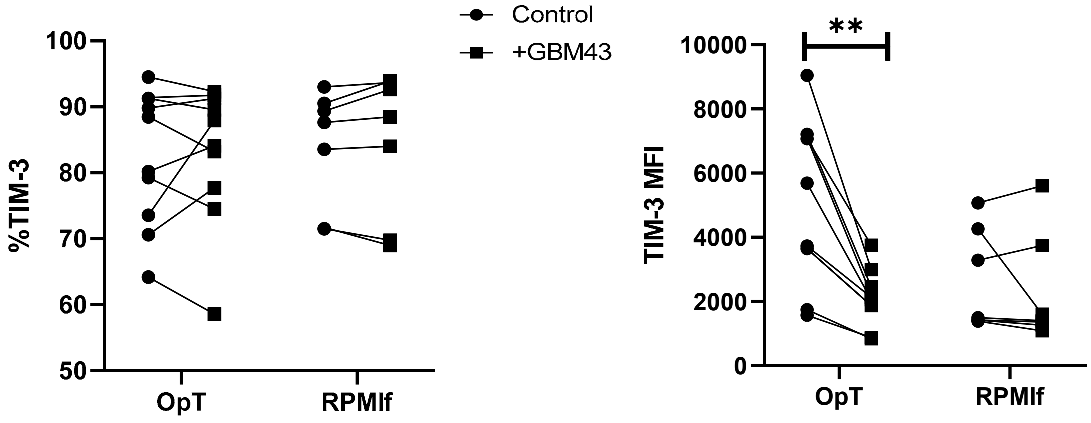

(B)

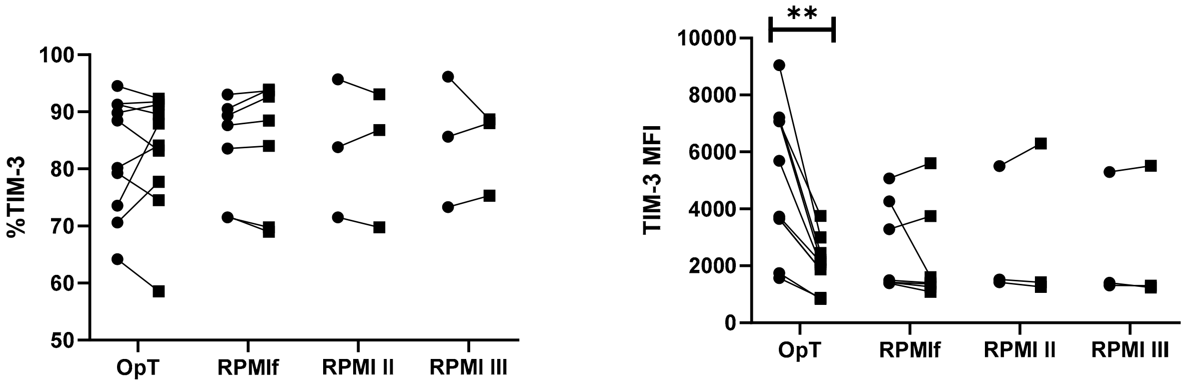

(C)

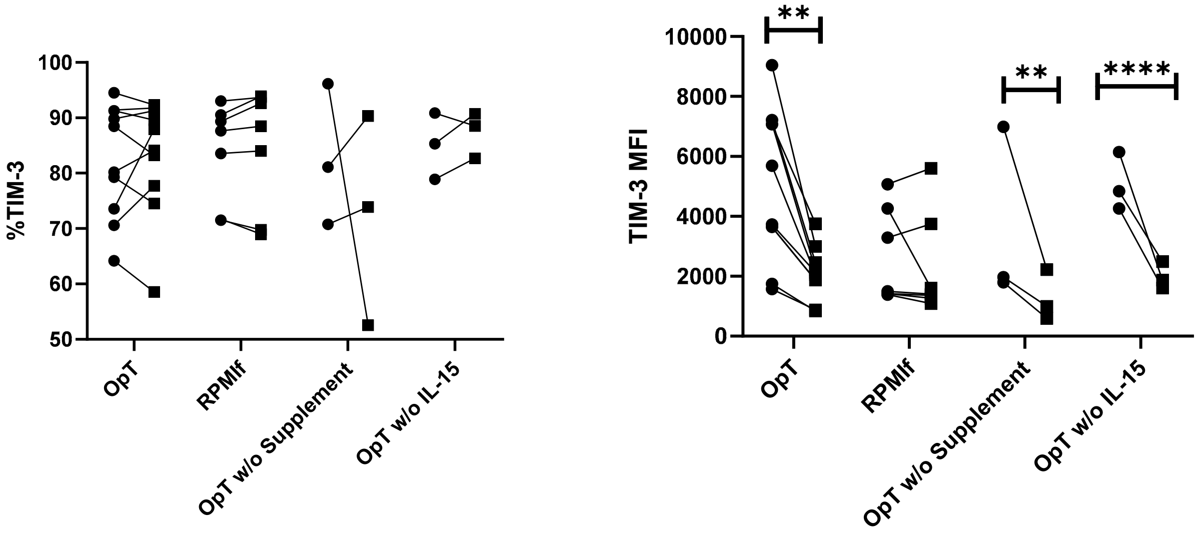

Figure S5. TIM-3 expression on human NK cells by individual donor. (A) Differences between OpTmizer™ (n = 10 donors) and RPMI media (n = 7); (B) Differences between RPMI media compositions (n = 3); (C) Differences between supplement compositions (n = 3). All stimulation conditions were consistent and as described in Materials and Methods.

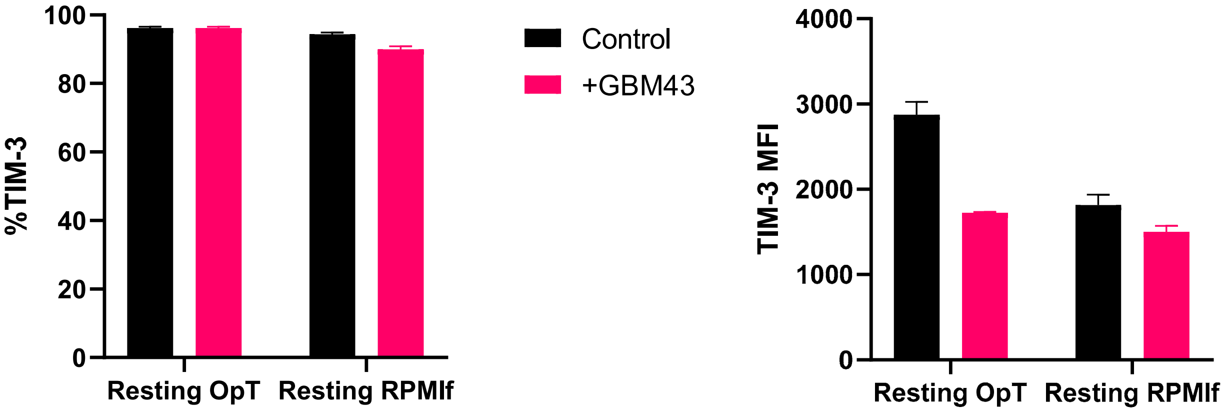

Figure S6. TIM-3 expression on resting human NK cells in response to GBM cells. TIM-3 percentage (left) and MFI (right) on resting NK cells upon co-culture with GBM43 cells at E:T 2.5:1 (n = 3 independent samples). NK cells were rested in culture media with no OpTmizer™ supplement or stimulation by cytokines for 24 hours prior to stimulation with GBM43 cells. All stimulation conditions were as described in Materials and Methods.

.

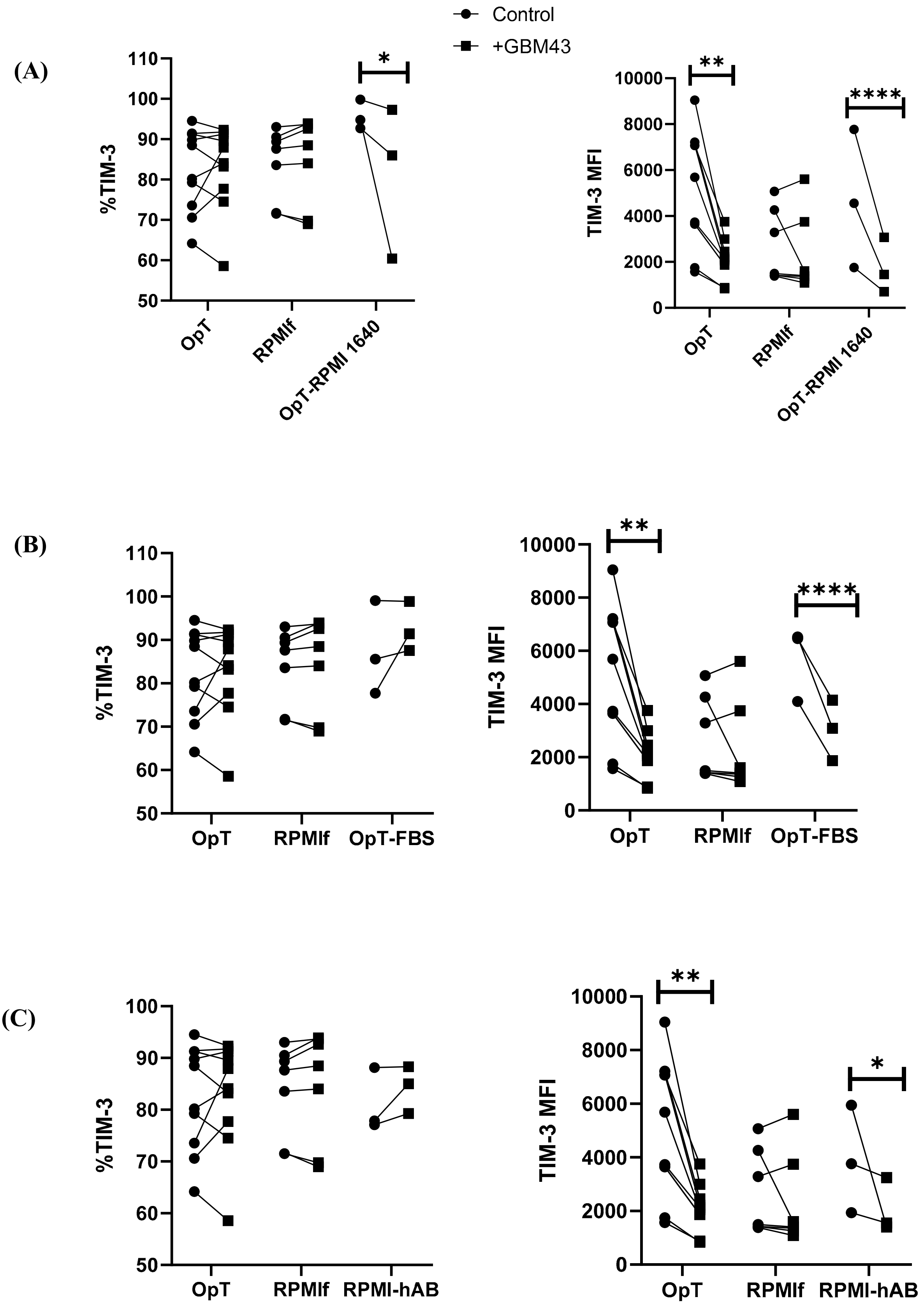

Figure S7. TIM-3 expression on NK cells by individual donor. (**A**) Differences against RPMI 1640 (n = 3 donors). (**B**) Differences against FBS (n = 3). (**C**) Differences against human AB serum (n = 3). All stimulation conditions were consistent and as described in *Materials and Methods*.

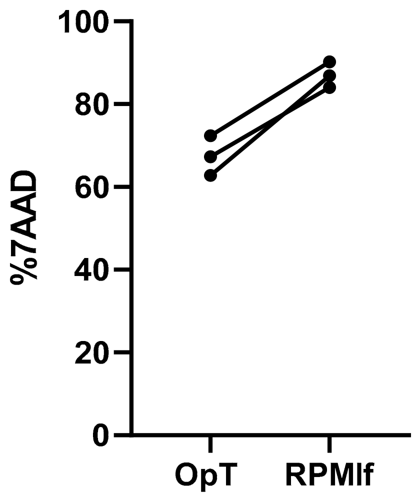

Figure S8. Cytotoxicity of NK cells against GBM43 cells in different media for individual donors. The killing assay was carried out over 4h incubation and detected via flow cytometric 7-ADD/CFSE staining as described in *Materials and Methods* (n = 3 donors).

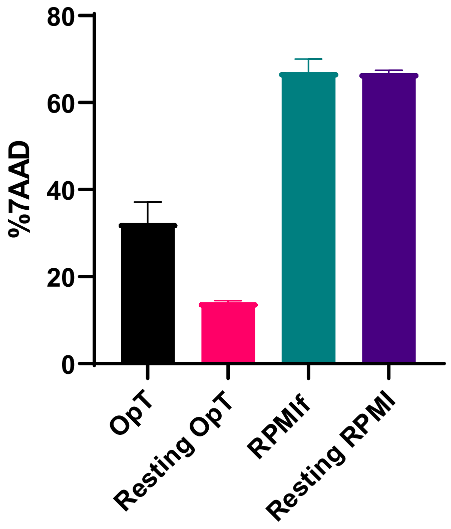

Figure S9. Effect of blockade of TIM-3 on the cytotoxicity of resting NK cells against GBM43 cells. The killing assay was carried out over 4h incubation and detected via flow cytometric 7-ADD/CFSE staining as described in *Materials and Methods*. NK cells were rested in medium with no supplementation or cytokines for 24 hours prior to being used in the killing assay (n = 3 independent samples).

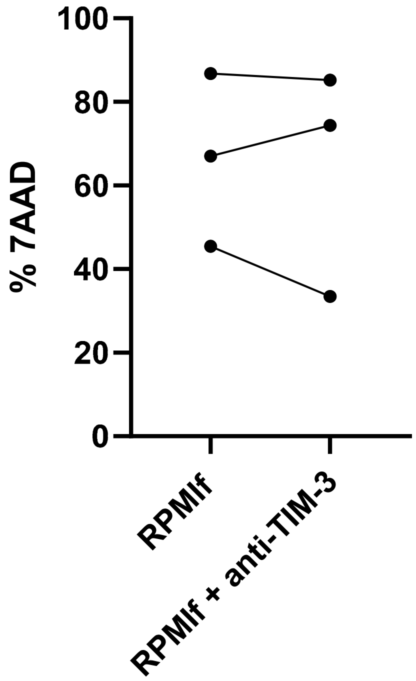

Figure S10. Effect of blockade of TIM-3 on the cytotoxicity of RPMIf-expanded NK cells against GBM43 cells for individual donors. The killing assay was carried out over 4h incubation and detected via flow cytometric 7-ADD/CFSE staining as described in *Materials and Methods* (n = 3 donors).

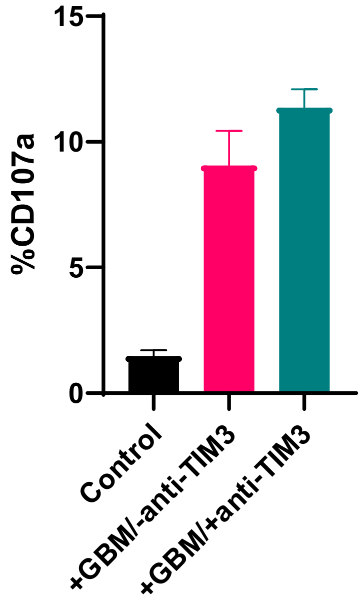

Figure S11. Effect of blockade of TIM-3 on the degranulation capacity of OpTmizer™-NK cells against GBM43 cells. The co-culture assay was carried out over 4h incubation and detected via flow cytometric staining of CD107a as described in *Materials and Methods* (n = 6 independent samples).

Table S2. Correlation of TIM-3 (*HAVCR2*) expression and NK cell presence in GBM based on bioinformatics analysis of TCGA patient data.

Pathway	pval	padj	ES	NES	Direction
NK_signature_custom	0.0727273	0.0727273	0.7964387	1.478208	Positive

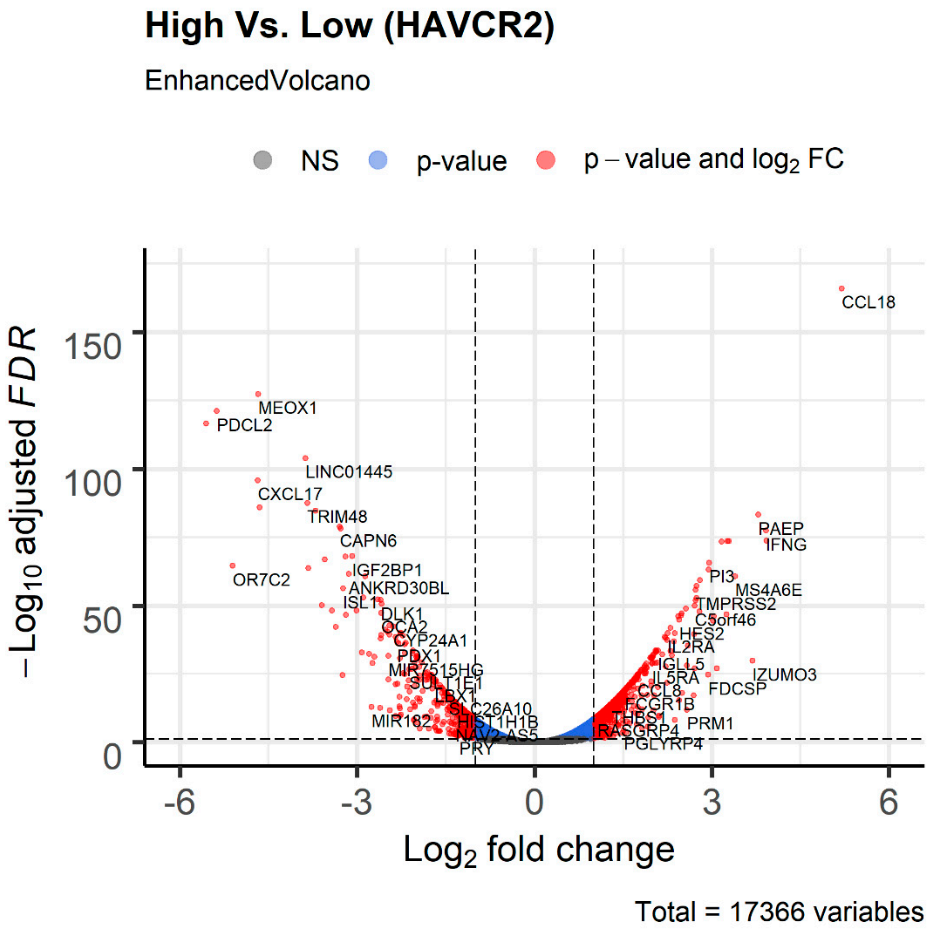

Figure S12. Volcano plot showing differentially expressed genes in *HAVCR2*⁺ GBM patient datasets. Top up-regulated and down-regulated genes are shown following stratification of GBM patient data based on high vs. low *HAVCR2* expression (N = 176). Bioinformatics analysis is described in the Materials and Methods section.

(A)

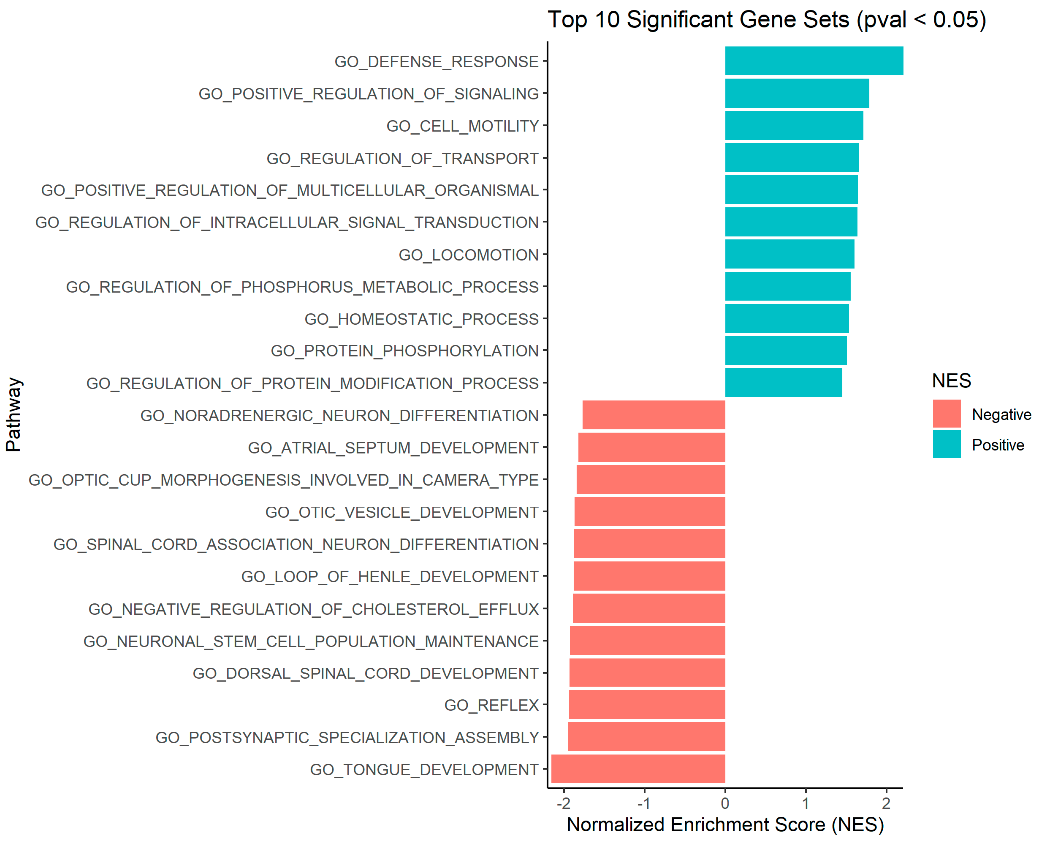

Top 10 Significant Gene Sets (pval < 0.05)
GO_DEFENSE_RESPONSE
GO_POSITIVE_REGULATION_OF_SIGNALING
GO_CELL_MOTILITY
GO_REGULATION_OF_TRANSPORT
GO_POSITIVE_REGULATION_OF_MULTICELLULAR_ORGANISMAL
GO_REGULATION_OF_INTRACELLULAR_SIGNAL_TRANSDUCTION
GO_LOCOMOTION
GO_REGULATION_OF_PHOSPHORUS_METABOLIC_PROCESS
GO_HOMEOSTATIC_PROCESS
GO_PROTEIN_PHOSPHORYLATION
GO_REGULATION_OF_PROTEIN_MODIFICATION_PROCESS
GO_NORADRENERGIC_NEURON_DIFFERENTIATION
GO_ATRIAL_SEPTUM_DEVELOPMENT
GO_OPTIC_CUP_MORPHOGENESIS_INVOLVED_IN_CAMERA_TYPE
GO_OTIC_VESICLE_DEVELOPMENT
GO_SPINAL_CORD_ASSOCIATION_NEURON_DIFFERENTIATION
GO_LOOP_OF_HENLE_DEVELOPMENT
GO_NEGATIVE_REGULATION_OF_CHOLESTEROL_EFFLUX
GO_NEURONAL_STEM_CELL_POPULATION_MAINTENANCE
GO_DORSAL_SPINAL_CORD_DEVELOPMENT
GO_REFLEX
GO_POSTSYNAPTIC_SPECIALIZATION_ASSEMBLY
GO_TONGUE_DEVELOPMENT
Pathway
-2
-1
0
1
2
Normalized Enrichment Score (NES)
NES
Negative
Positive

(B)

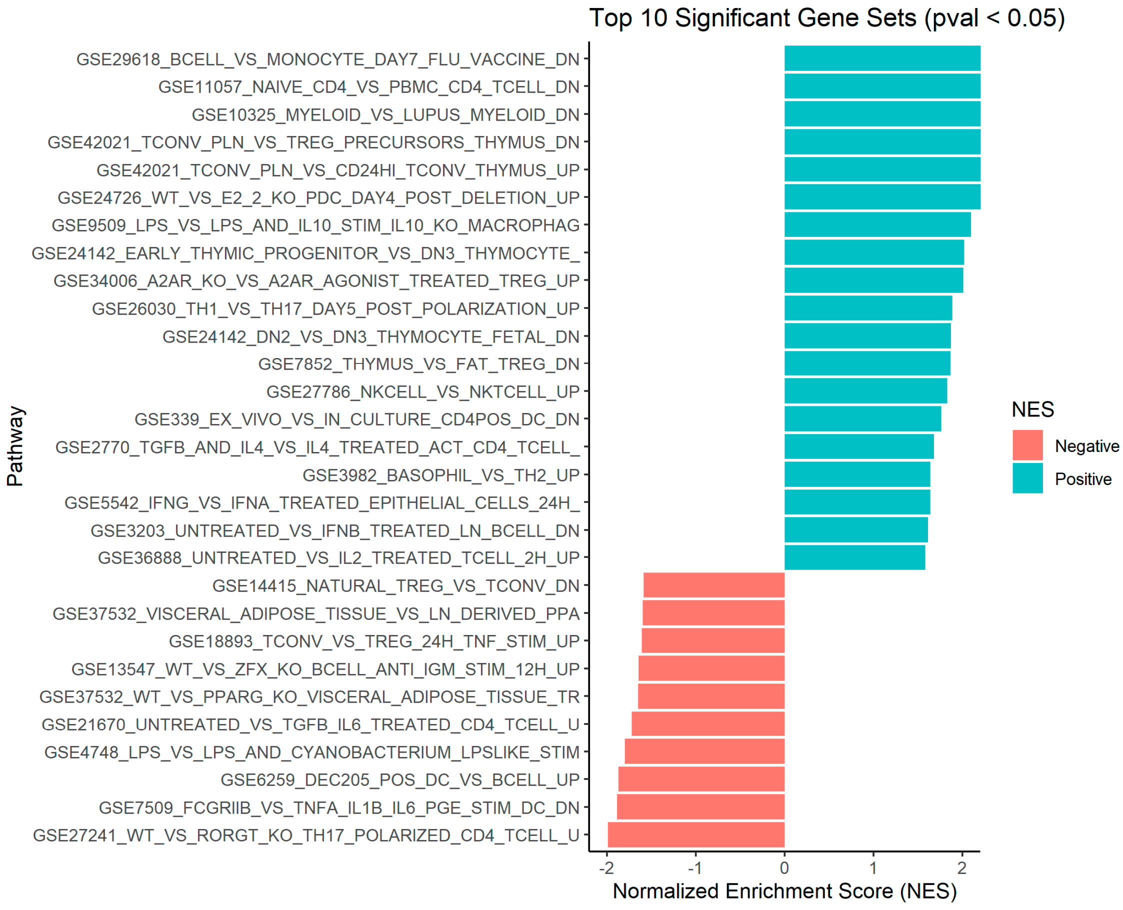

Top 10 Significant Gene Sets (pval < 0.05)
GSE29618_BCELL_VS_MONOCYTE_DAY7_FLU_VACCINE_DN
GSE11057_NAIVE_CD4_VS_PBMC_CD4_TCELL_DN
GSE10325_MYELOID_VS_LUPUS_MYELOID_DN
GSE42021_TCONV_PLN_VS_TREG_PRECURSORS_THYMUS_DN
GSE42021_TCONV_PLN_VS_CD24HI_TCONV_THYMUS_UP
GSE24726_WT_VS_E2_2_KO_PDC_DAY4_POST_DELETION_UP
GSE9509_LPS_VS_LPS_AND_IL10_STIM_IL10_KO_MACROPHAG
GSE24142_EARLY_THYMIC_PROGENITOR_VS_DN3_THYMOCYTE_
GSE34006_A2AR_KO_VS_A2AR_AGONIST_TREATED_TREG_UP
GSE26030_TH1_VS_TH17_DAY5_POST_POLARIZATION_UP
GSE24142_DN2_VS_DN3_THYMOCYTE_FETAL_DN
GSE7852_THYMUS_VS_FAT_TREG_DN
GSE27786_NKCELL_VS_NKTCELL_UP
GSE339_EX_VIVO_VS_IN_CULTURE_CD4POS_DC_DN
GSE2770_TGFB_AND_IL4_VS_IL4_TREATED_ACT_CD4_TCELL_
GSE3982_BASOPHIL_VS_TH2_UP
GSE5542_IFNG_VS_IFNA_TREATED_EPITHELIAL_CELLS_24H_
GSE3203_UNTREATED_VS_IFNB_TREATED_LN_BCELL_DN
GSE36888_UNTREATED_VS_IL2_TREATED_TCELL_2H_UP
GSE14415_NATURAL_TREG_VS_TCONV_DN
GSE37532_VISCERAL_ADIPOSE_TISSUE_VS_LN_DERIVED_PPA
GSE18893_TCONV_VS_TREG_24H_TNF_STIM_UP
GSE13547_WT_VS_ZFX_KO_BCELL_ANTI_IGM_STIM_12H_UP
GSE37532_WT_VS_PPARG_KO_VISCERAL_ADIPOSE_TISSUE_TR
GSE21670_UNTREATED_VS_TGFB_IL6_TREATED_CD4_TCELL_U
GSE4748_LPS_VS_LPS_AND_CYANOBACTERIUM_LPSLIKE_STIM
GSE6259_DEC205_POS_DC_VS_BCELL_UP
GSE7509_FCGRIIB_VS_TNFA_IL1B_IL6_PGE_STIM_DC_DN
GSE27241_WT_VS_RORGT_KO_TH17_POLARIZED_CD4_TCELL_U
Pathway
-2
-1
0
1
2
Normalized Enrichment Score (NES)
NES
Negative
Positive

Figure S13. GSEA analysis of top up- and down-regulated genes in *HAVCR2*⁺ GBM patient samples (TCGA). (**A**) GSEA analysis with GO-Biological Processes collection in MSigDB carried out on *HAVCR2*⁺ GBM patient samples; (**B**) GSEA analysis with Immunologic collection in MSigDB carried out on *HAVCR2*⁺ GBM patient samples.

(A)

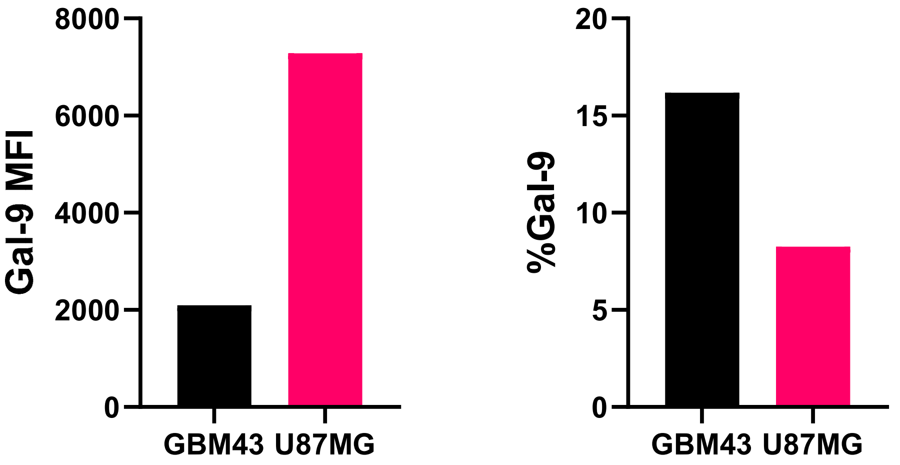

(B)

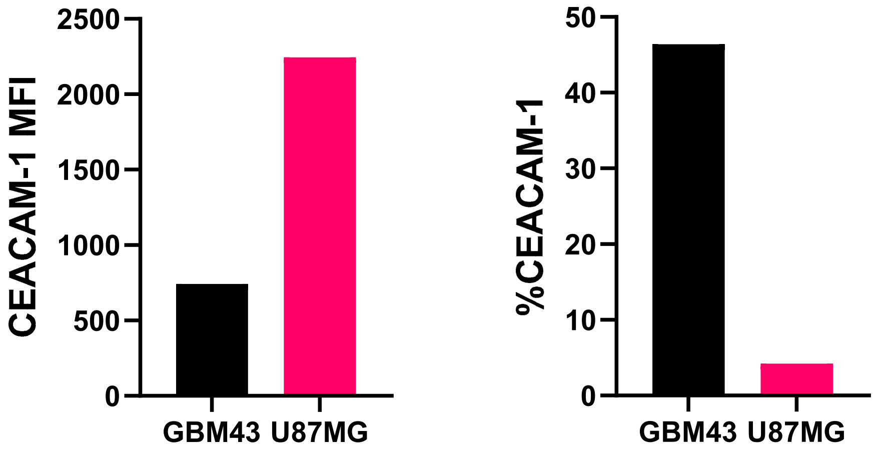

Figure S14. TIM-3 ligand expression on cancer cells. (**A**) Galectin-9 percentage (left panel) and MFI (right panel) on GBM34 and U87MG glioblastoma cells; (**B**) CEACAM-1 percentage (left panel) and MFI (right panel) on GBM34 and U87MG glioblastoma cells.

(A)

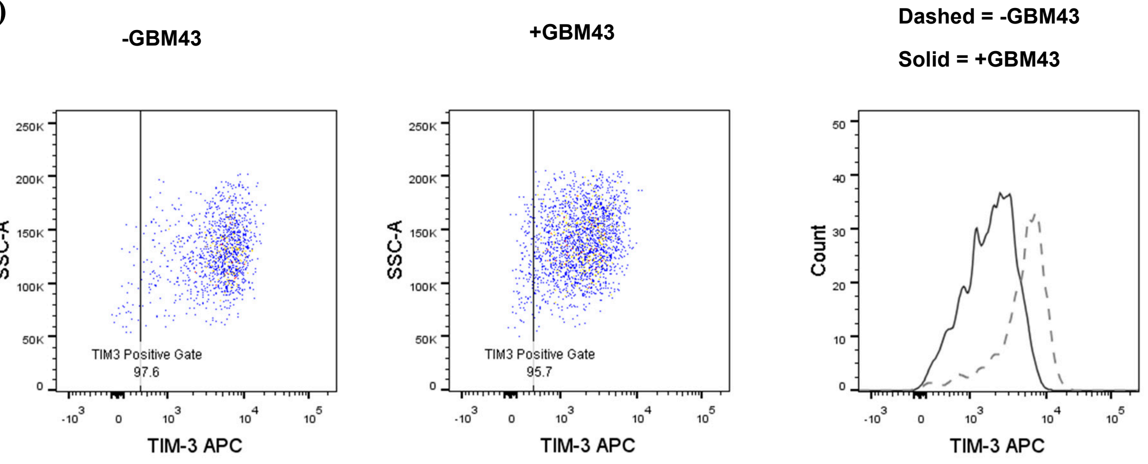

(B)

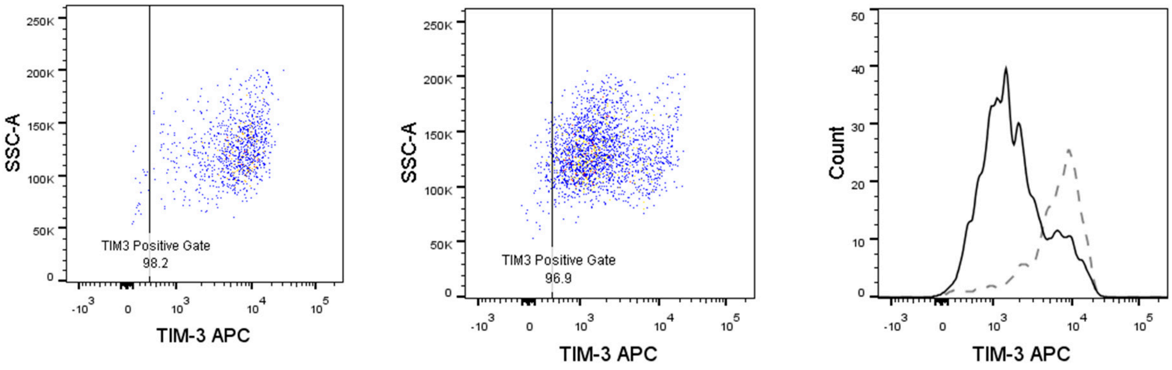

Figure S15. Flow cytometry dot plots for TIM-3 expression on human NK cells. Expression of TIM-3 on NK cells stimulated in (**A**) OpTmizer™ medium without (left panel) and in the presence (right panel) of GBM43 cell at E:T 2.5:1, and (**B**) RPMIf medium without (left panel) and in the presence (right panel) of GBM43 cells at E:T 2.5:1. All stimulation conditions were as described in Materials and Methods.

Table S3. MESF values for MFI conditions recorded in the manuscript representing TIM-3 expression on NK cells. Values were converted to MESF units using APC-conjugated MESF beads using QuickCal® v2.3 software.

Conditions	MFI	MESF
OpT, PNK + GBM43	831.667	40817.6
OpT, PNK only	1173.67	65951.6
RPMIf, PNK + GBM43	1134.67	61220.2
RPMIf, PNK only	1128.67	60796.2

© 2020 by the authors. Licensee MDPI, Basel, Switzerland. This article is an open access article distributed under the terms and conditions of the Creative Commons Attribution (CC BY) license (http://creativecommons.org/licenses/by/4.0/).
